# Defining Polysaccharide-Specific Antibody Targets against Vibrio cholerae O139 in Humans following O139 Cholera and following Vaccination with a Commercial Bivalent Oral Cholera Vaccine, and Evaluation of Conjugate Vaccines Targeting O139

**DOI:** 10.1128/mSphere.00114-21

**Published:** 2021-07-07

**Authors:** Mohammad Kamruzzaman, Meagan Kelly, Richelle C. Charles, Jason B. Harris, Stephen B. Calderwood, Aklima Akter, Rajib Biswas, M. Hasanul Kaisar, Taufiqur R. Bhuiyan, Louise C. Ivers, Ralph Ternier, Jean-Gregory Jerome, Hélene B. Pfister, Xiaowei Lu, Sameh E. Soliman, Bart Ruttens, Rina Saksena, Jana Mečárová, Alžbeta Čížová, Firdausi Qadri, Slavomír Bystrický, Pavol Kováč, Peng Xu, Edward T. Ryan

**Affiliations:** a Division of Infectious Diseases, Massachusetts General Hospital, Boston, Massachusetts, USA; b International Centre for Diarrhoeal Disease Research Bangladesh, Dhaka, Bangladesh; c Department of Medicine, Harvard Medical School, Boston, Massachusetts, USA; d Department of Pediatrics, Harvard Medical School, Boston, Massachusetts, USA; e Division of Global Health, MassGeneral Hospital for Children, Boston, Massachusetts, USA; f Department of Global Health and Social Medicine, Harvard Medical School, Boston, Massachusetts, USA; g Partners In Health/Zanmi Lasante, Cange, Haiti; h NIDDK, LBC, National Institutes of Health, Bethesda, Maryland, USA; i Institute of Chemistry, Slovak Academy of Sciences, Bratislava, Slovak Republic; j Department of Immunology and Infectious Diseases, Harvard T.H. Chan School of Public Health, Boston, Massachusetts, USA; University of Florida

**Keywords:** *Vibrio cholerae* O139, O-specific polysaccharide, synthetic saccharides, conjugate vaccine, immune response, immunity

## Abstract

Cholera caused by Vibrio cholerae O139 could reemerge, and proactive development of an effective O139 vaccine would be prudent. To define immunoreactive and potentially immunogenic carbohydrate targets of Vibrio cholerae O139, we assessed immunoreactivities of various O-specific polysaccharide (OSP)-related saccharides with plasma from humans hospitalized with cholera caused by O139, comparing responses to those induced in recipients of a commercial oral whole-cell killed bivalent (O1 and O139) cholera vaccine (WC-O1/O139). We also assessed conjugate vaccines containing selected subsets of these saccharides for their ability to induce protective immunity using a mouse model of cholera. We found that patients with wild-type O139 cholera develop IgM, IgA, and IgG immune responses against O139 OSP and many of its fragments, but we were able to detect only a moderate IgM response to purified O139 OSP-core, and none to its fragments, in immunologically naive recipients of WC-O1/O139. We found that immunoreactivity of O139-specific polysaccharides with antibodies elicited by wild-type infection markedly increase when saccharides contain colitose and phosphate residues, that a synthetic terminal tetrasaccharide fragment of OSP is more immunoreactive and protectively immunogenic than complete OSP, that native OSP-core is a better protective immunogen than the synthetic OSP lacking core, and that functional vibriocidal activity of antibodies predicts *in vivo* protection in our model but depends on capsule thickness. Our results suggest that O139 OSP-specific responses are not prominent following vaccination with a currently available oral cholera vaccine in immunologically naive humans and that vaccines targeting V. cholerae O139 should be based on native OSP-core or terminal tetrasaccharide.

**IMPORTANCE** Cholera is a severe dehydrating illness of humans caused by Vibrio cholerae serogroup O1 or O139. Protection against cholera is serogroup specific, and serogroup specificity is defined by O-specific polysaccharide (OSP). Little is known about immunity to O139 OSP. In this study, we used synthetic fragments of the O139 OSP to define immune responses to OSP in humans recovering from cholera caused by V. cholerae O139, compared these responses to those induced by the available O139 vaccine, and evaluated O139 fragments in next-generation conjugate vaccines. We found that the terminal tetrasaccharide of O139 is a primary immune target but that the currently available bivalent cholera vaccine poorly induces an anti-O139 OSP response in immunologically naive individuals.

## INTRODUCTION

Cholera is a severe dehydrating diarrheal disease of humans that can be caused by Vibrio cholerae serogroup O1 or O139 organisms (1–3). Although O139 has at present disappeared as a cause of cholera globally (4, 5), epidemic cholera caused by O139 could reemerge (6) and proactive development of vaccines protective against O139 would be prudent. At present, the only commercially available cholera vaccine against V. cholerae O139 is a bivalent oral cholera vaccine (WC-O1/O139) that contains killed V. cholerae O1 and killed O139 organisms (7, 8). The bivalent vaccine induces significantly lower immune responses to the O139 component of the vaccine than to the O1 component (9), and although the vaccine has been shown to be protective against cholera caused by V. cholerae O1, it has never been evaluated for protective efficacy against V. cholerae O139 (8). Protection against cholera is serogroup specific, the specificity being defined by the O-specific polysaccharide (OSP) of V. cholerae (10, 11). Previous infection with V. cholerae O1 does not provide protection against O139, and vice versa (10). The OSPs of V. cholerae O1 and O139 are distinct, although core oligosaccharides are identical (12–15). V. cholerae O139 is also encapsulated, while V. cholerae O1 is not (16). The capsule of O139, which is not directly attached to the bacterium, is a polysaccharide whose repeating unit is the same as that of V. cholerae O139 OSP (13, 16). Capsule thickness varies by V. cholerae O139 strain (17). O139 capsule surrounds lipopolysaccharide (LPS), which includes O139 OSP (a hexasaccharide) as its upstream terminal epitope. The structure of that hexasaccharide and the mode of its attachment to the core oligosaccharide, to form OSP-core (OSPc), as well as structural details in the core oligosaccharide have recently been confirmed (18). Unfortunately, little is known about polysaccharide-specific immune responses to V. cholerae O139, despite the potential significance of such information since protection against cholera is serogroup specific.

Our objective in this study, therefore, was to define the polysaccharide immune targets against V. cholerae O139 in humans recovering from cholera caused by V. cholerae O139, to compare these responses to those in WC-O1/O139 recipients, and to evaluate potential next-generation carbohydrate-based conjugate vaccines against O139. To do this, we made conjugates from OSP-core isolated from wild-type V. cholerae O139, a synthetic V. cholerae O139 OSP (hexasaccharide), and fragments and derivatives of the latter, using bovine serum albumin (BSA) or a recombinant protein (rTTHc) as the carrier. rTTHc is a nontoxic 52-kDa recombinant tetanus toxoid heavy chain fragment (19, 20). We assessed immunoreactivity of BSA-based conjugates using convalescent-phase plasma from humans who recovered from O139 cholera, comparing responses to those in matched WC-O1/O139 recipients, and evaluated, in mice, a selected subset of rTTHc-based conjugates as potential vaccine candidates against disease caused by Vibrio cholerae O139.

## RESULTS

### Immunoreactivity of BSA conjugates with plasma from humans recovering from O139 cholera and recipients of WC-O1/O139.

**FIG 1:**
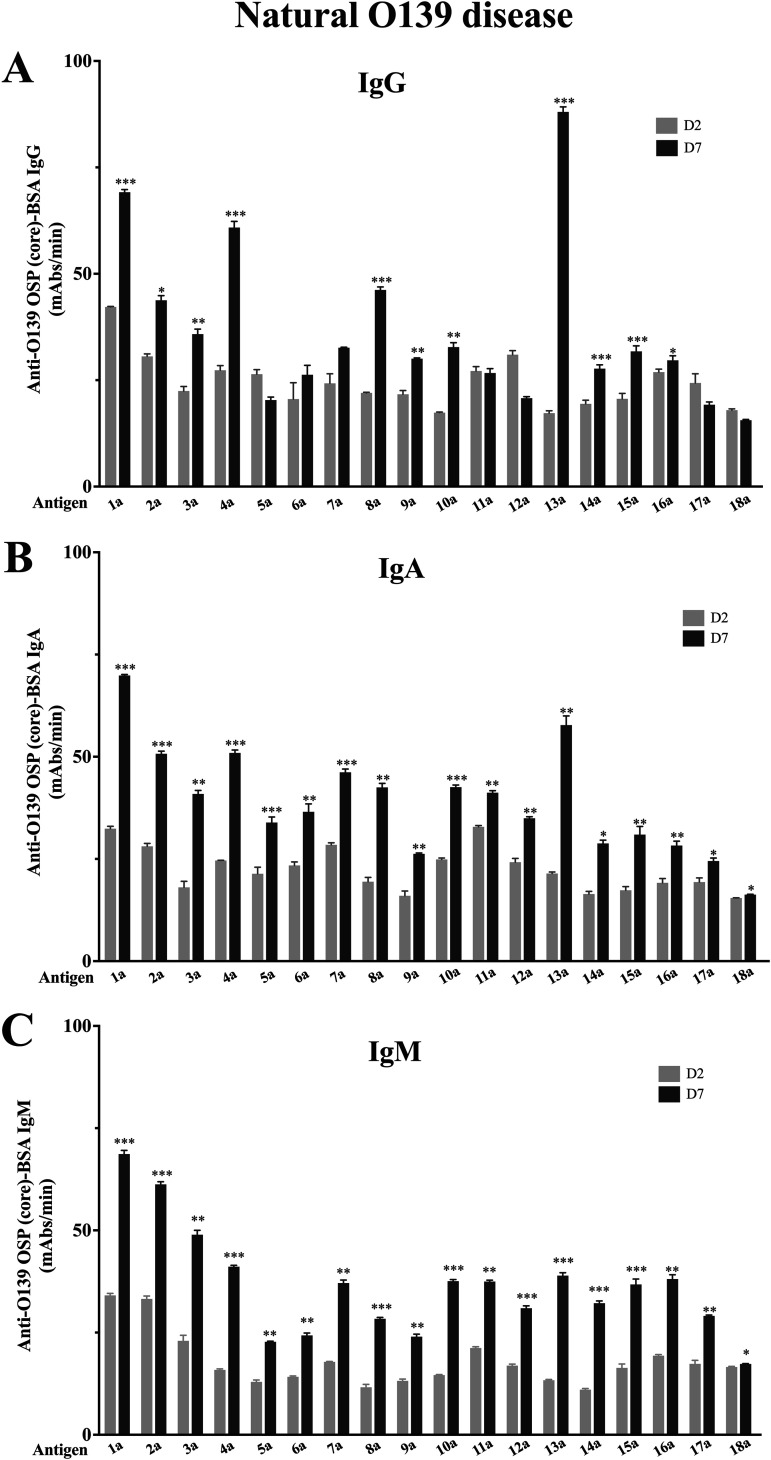
Immunoreactivity of plasma from humans with O139 cholera in Bangladesh against O139 OSPc and synthetic fragments conjugated to BSA. Immunoreactivities (IgG [A], IgA [B], and IgM [C]) of different preparations of native OSP-core and synthetic O139 OSP and fragments conjugated to BSA were measured in acute-phase (day 2) and convalescent-phase (day 7) plasma of patients with O139 cholera in Dhaka, Bangladesh. *, statistically significant difference (*P* ≤ 0.05) from the baseline (day 2) response; **, *P* ≤ 0.01; ***, *P* ≤ 0.001. Conjugate 1a is O139 OSP-core (crude):BSA, conjugate 2a is O139 OSP-core (intermediate):BSA, conjugate 3a is O139 OSP-core (pure):BSA, and conjugates 4a to 18a are synthetic BSA conjugates (see Table 1 for details). mAbs, milli-absorbance units.

**FIG 2:**
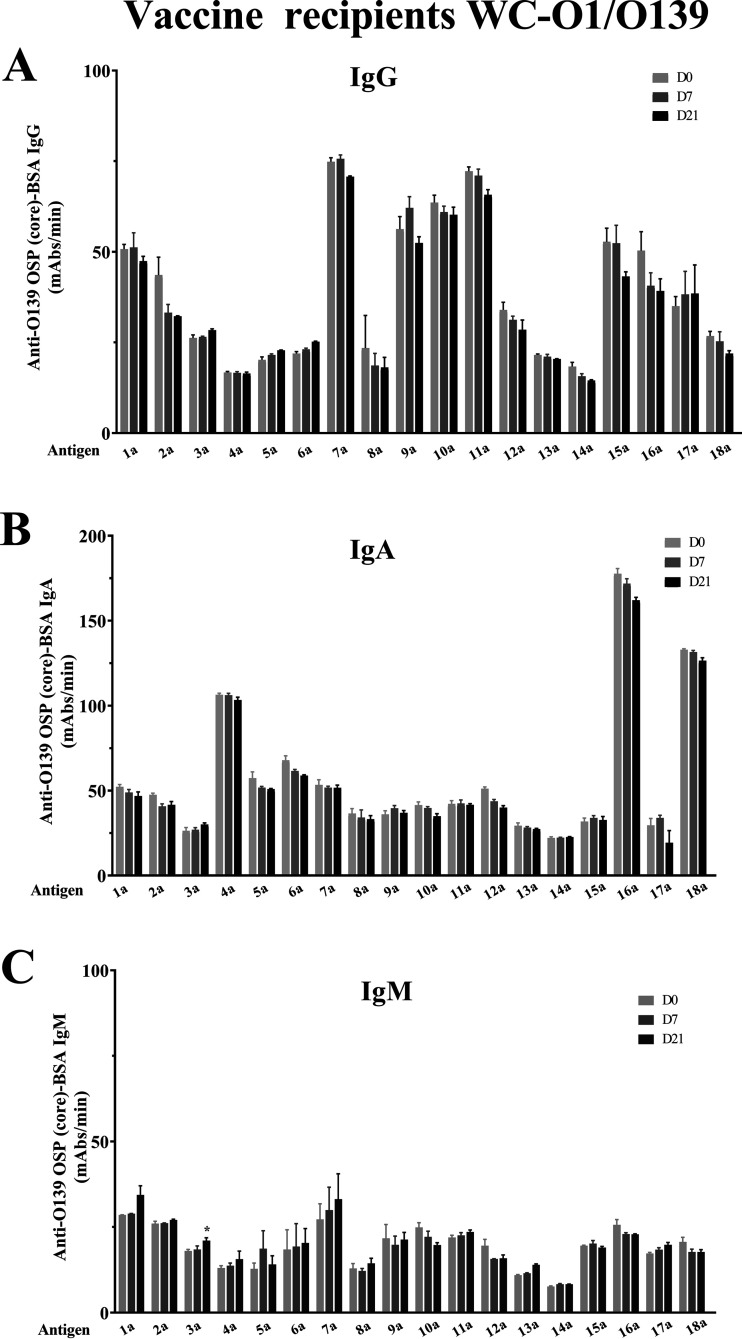
Immunoreactivity of plasma from humans vaccinated with WC-O1/O139 in Haiti against O139 OSPc and synthetic fragments conjugated to BSA. Immunoreactivities (IgG [A], IgA [B], and IgM [C]) of different preparations of native OSP-core and synthetic O139 OSP and fragments conjugated to BSA were measured in the acute phase (day 0) versus 7 days after day 0 dose (day 7) versus 7 days after day 14 vaccine dose (day 21) in Haitian vaccine recipients matched by age, sex, and blood group to the Bangladeshi patients depicted in Fig. 1. *, statistically significant difference (*P *≤ 0.05) from the baseline (day 2) response. For conjugate descriptions, see the legend to Fig. 1. mAbs, milli-absorbance units.

We assessed immunoreactivities of different native V. cholerae O139–OSP-core conjugates (conjugates 1a to 3a [Table 1]), analyzing IgM, IgA, and IgG isotype responses in humans recovering from cholera caused by V. cholerae O139 in Bangladesh (conjugates 1a, 2a, and 3a [Fig. 1]). We found the lowest baseline immunoreactivity, as defined by responses in acute-phase blood, with the pure preparation of native OSP-core (conjugate 3a). However, fold changes of immunoreactivity from day 7 to day 2 of crude (conjugate 1a), intermediate (conjugate 2a), and pure OSP-core preparation (conjugate 3a) were comparable. In comparison, we found no increase in any O139 OSP-specific IgA or IgG response in human recipients of WC-O1/O139, either 7 days after the first dose or 7 days after the second dose (Fig. 2A and B). We did detect a small increase in IgM immunoreactivity to pure OSP-core (conjugate 3a; *P* < 0.05 [Fig. 2C]) on day 21 following two doses of WC-O1/O139 compared to day 0 and a trend for increased IgM immunoreactivity on day 21 to crude OSP-core (conjugate 1a; *P* = 0.08 [Fig. 2C]). Since we found that humans surviving O139 cholera developed significant IgM, IgA, and IgG responses to all three native OSP-core preparations, we focused subsequent evaluations on native OSP-core using either the crude or pure preparations.

**TABLE 1:**
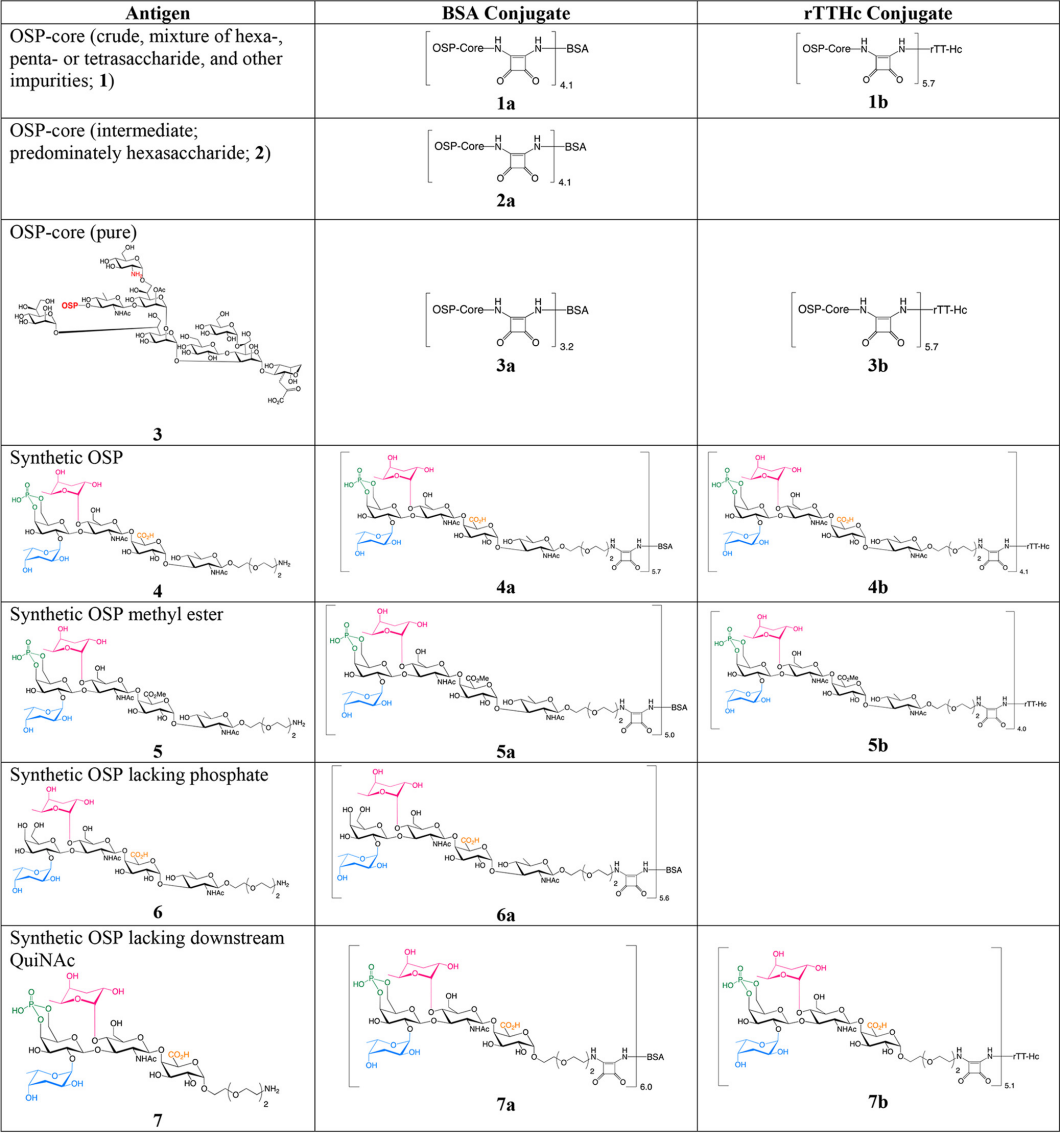

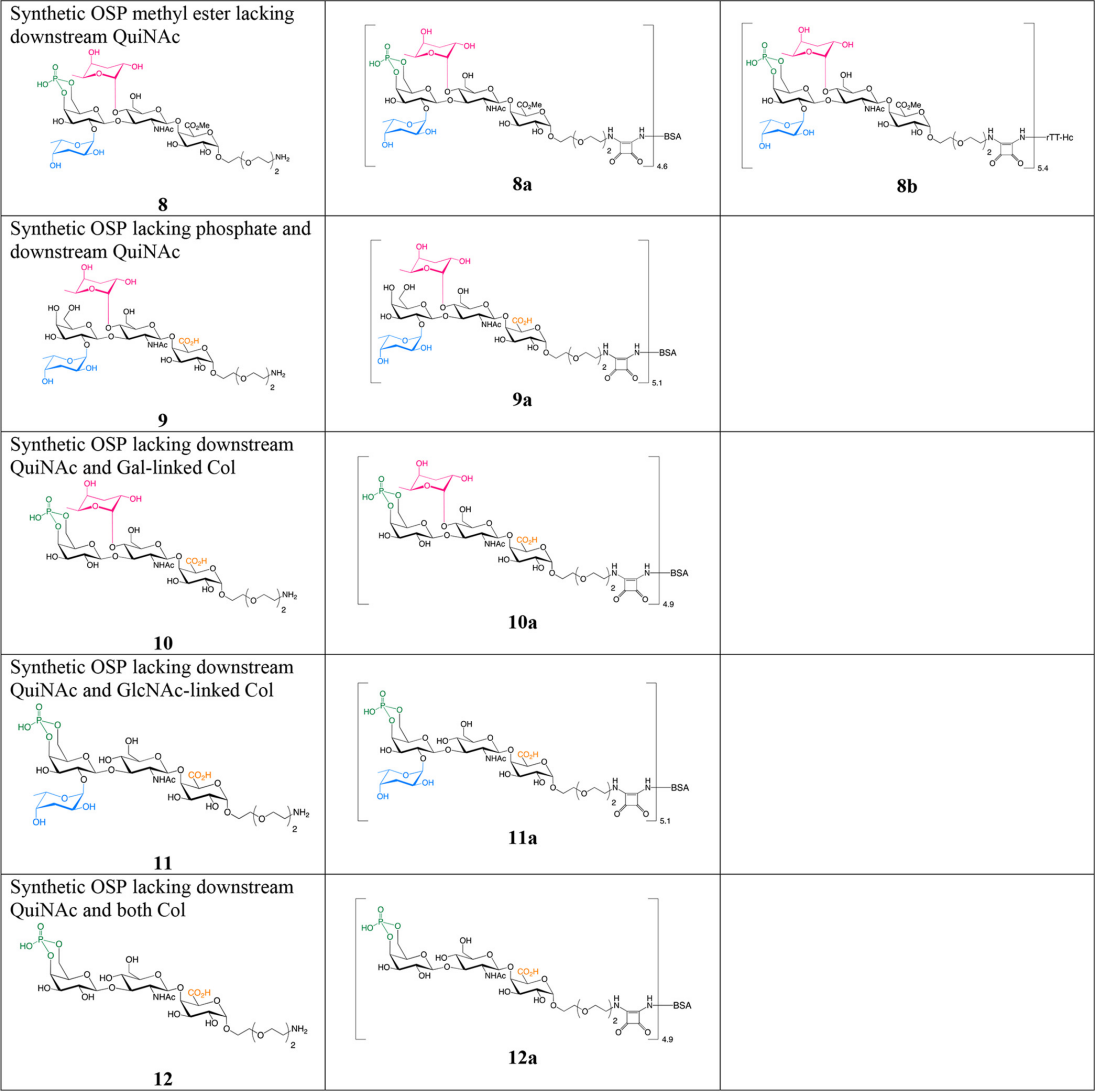

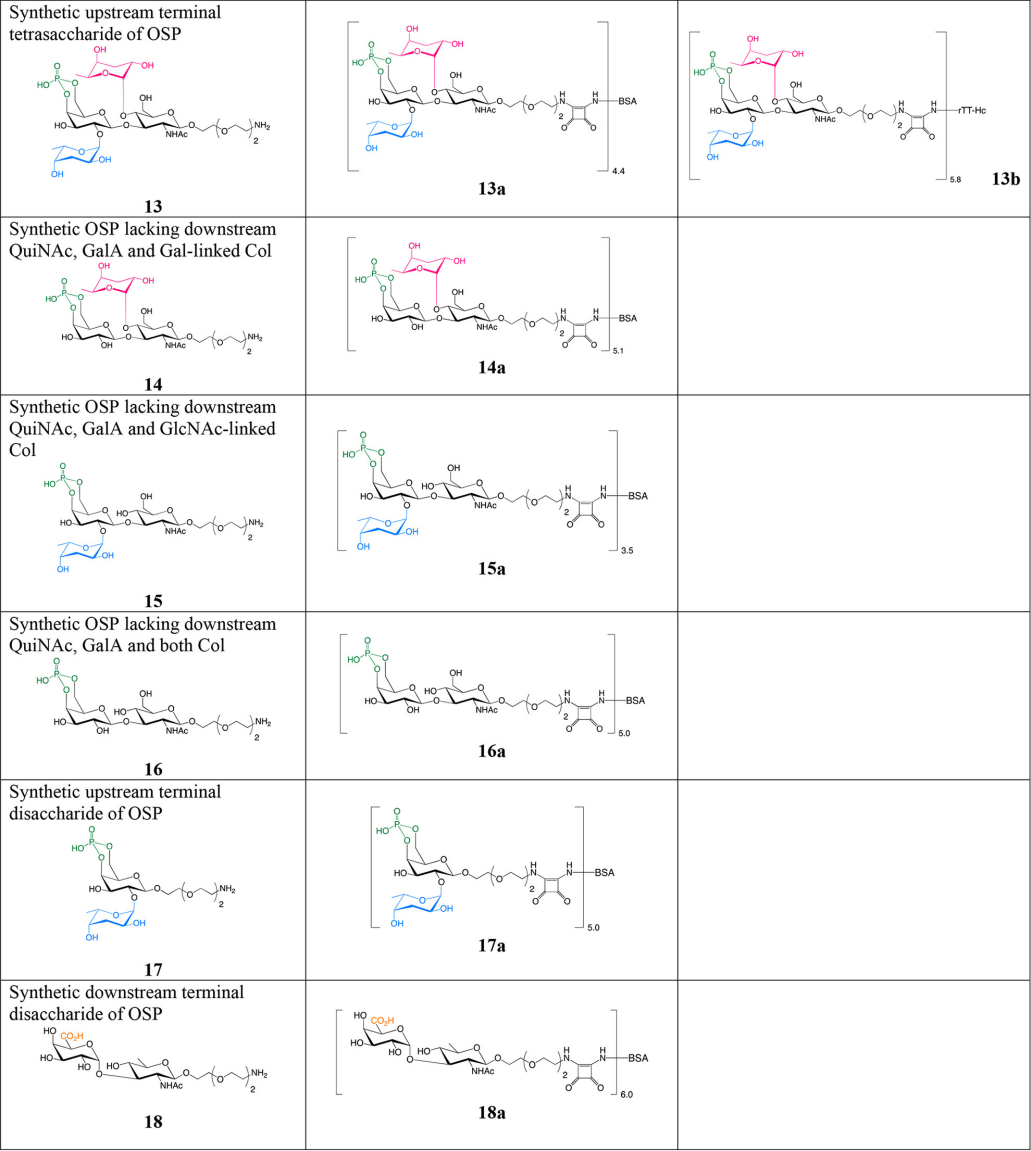
Structures of V. cholerae O139 antigens and conjugates used in this study^*a*^

a Numbers in lower right outside parentheses reflect the molar ratio of sugar to carrier protein in a conjugate. For preparation of the conjugates, see the supplemental material.

We also assessed immunoreactivities to conjugates of the synthetic OSP and its fragments and derivatives (conjugates 4a to 18a [Table 1]) of sera from patients surviving naturally acquired O139 cholera and WC-O1/O139 vaccine recipients (Fig. 1 and 2). In surviving patients, we found an increase in immunoreactivity in convalescence compared to acute phase of infection for all evaluated fragments and derivatives when assessing across all three antibody isotype responses. Increases in IgM and IgA responses were detectable against all saccharide preparations, with significant IgG responses detectable against a subset of preparations. Analysis of immunoreactivity of each sized fragment disclosed that removal of either one or both colitose (Col) units or the phosphate significantly decreased immunoreactivity. As opposed to IgM responses that demonstrated broad reactivity to the various preparations, more prominent IgA and IgG fold change responses were present for certain antigens, especially tetrasaccharide (conjugate 13a [Fig. 1A and B]; IgA *P* < 0.001; IgG *P* < 0.001). In comparison, we found no increase in immunoreactivity to any saccharide preparation other than native OSP-core IgM responses in vaccine recipients of WC-O1/O139 (Fig. 2).

### Assessing antigen-specific O139 conjugate vaccine candidates in mice.

**FIG 3:**
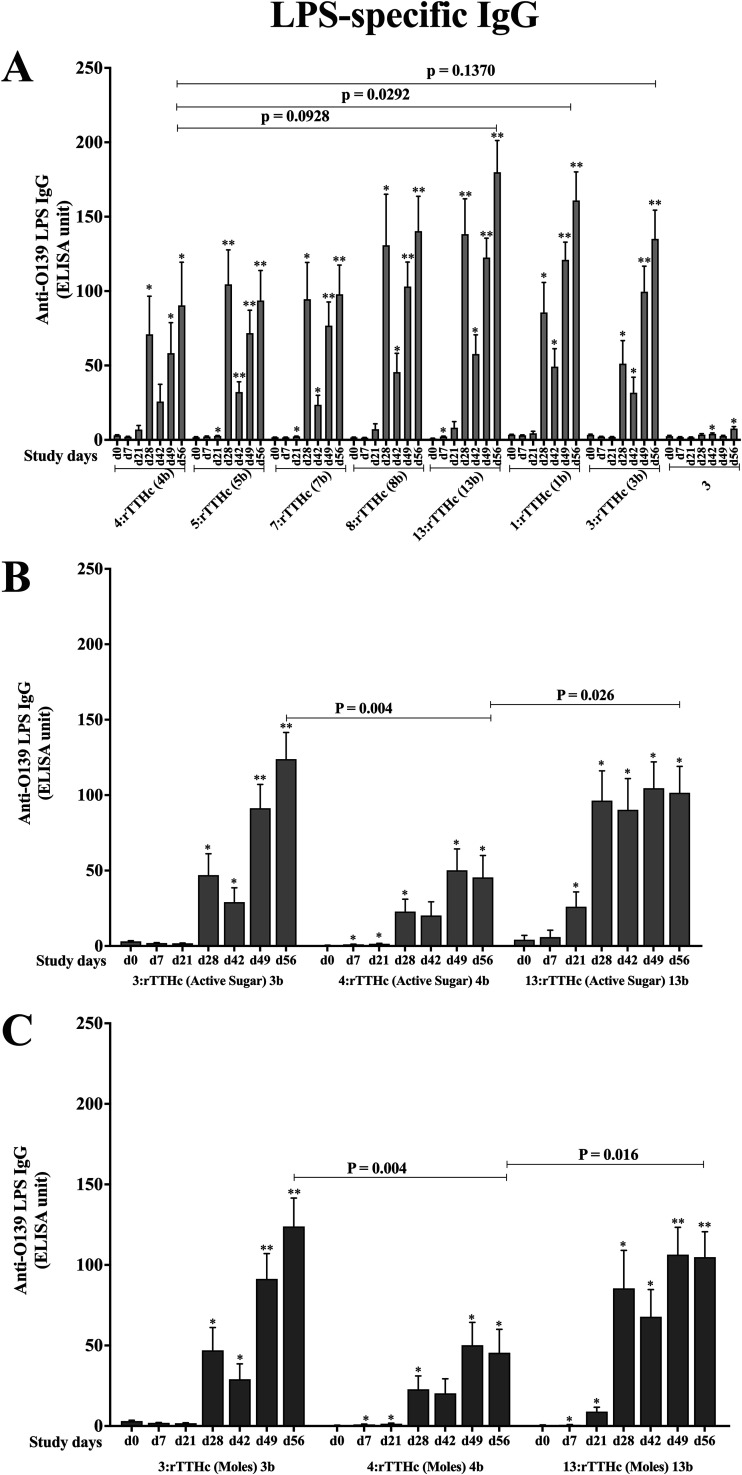
LPS-specific serum anti-O139 IgG responses in mice intramuscularly immunized with different fragments of OSP conjugated with rTTHc and unconjugated native pure OSP (preparation 3) equalized to total micrograms of saccharide (A), native OSPc (pure):rTTHc (conjugate 3b), synthetic OSP:rTTHc (conjugate 4b), and synthetic tetrasaccharide:rTTHc (conjugate 13b) equalized to mass of active sugar component in micrograms (lacking core and linker) (B), and conjugates 3b, 4b, and 13b equalized to moles of saccharide (C). Means and standard errors of the means are reported for each group. *, statistically significant difference (*P* < 0.05) from baseline (day 0) titer; **, *P* ≤ 0.01.

**FIG 4:**
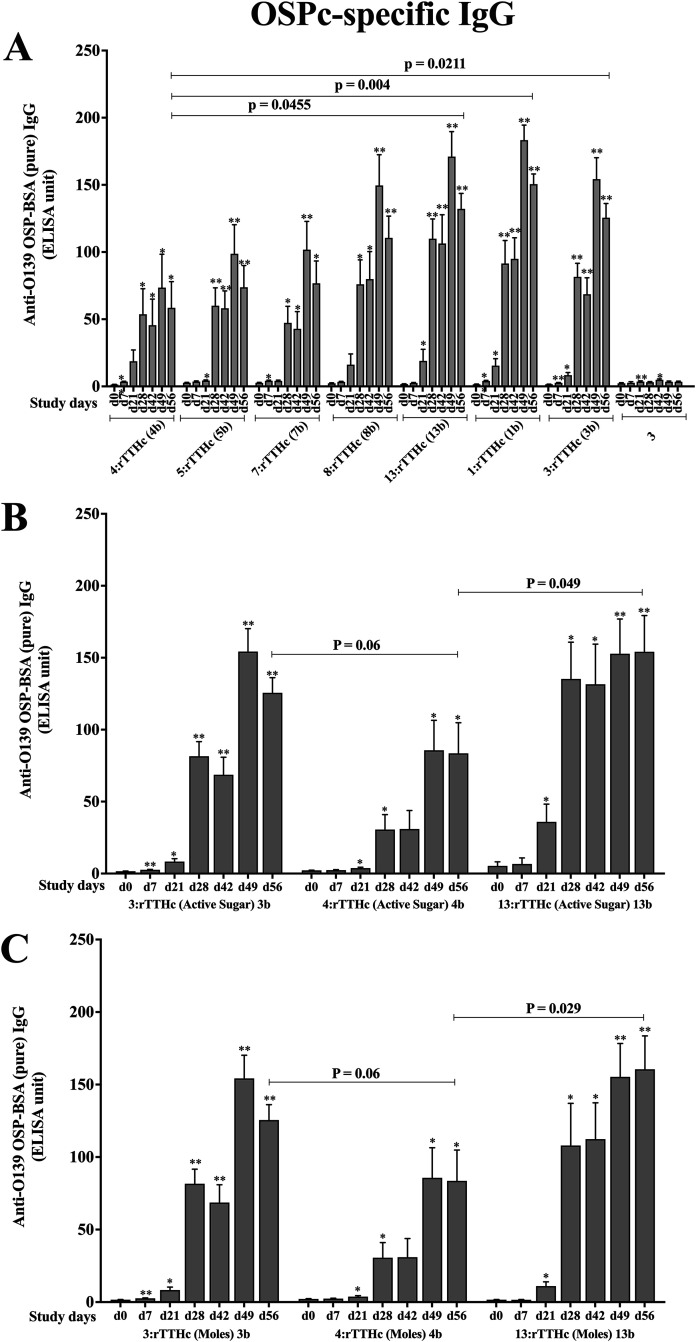
OSPc:BSA-specific serum anti-O139 IgG responses in mice intramuscularly immunized with (A) different fragments of OSP conjugated with rTTHc and unconjugated native pure OSP (preparation 3) equalized to total micrograms of saccharide (A), native OSPc (pure):rTTHc (conjugate 3b), synthetic OSP:rTTHc (conjugate 4b), and synthetic tetrasaccharide:rTTHc (conjugate 13b) equalized to mass of active sugar component in micrograms (lacking core and linker) (B), and conjugates 3b, 4b, and 13b equalized to moles of saccharide (C). Means and standard errors of the means are reported for each group. *, statistically significant difference (*P* < 0.05) from baseline (day 0) titer; **, *P* ≤ 0.01.

To further judge immune responses to O139 antigens, we assessed immunogenicity to a subset of O139 saccharides conjugated to protein carrier rTTHc. We assessed conjugates containing synthetic OSP (hexasaccharide), its pentasaccharide and tetrasaccharide fragments, or native OSP-core (conjugates 1b, 3b, 4b, 5b, 7b, 8b, and 13b [Table 1]). As assessed by their ability to induce LPS-specific IgG responses (Fig. 3), all conjugates were immunogenic following vaccination equalized by mass of total saccharide. Boosting of immune responses was observed following the 2nd and 3rd vaccinations, and then there was a subsequent increase in late convalescent-phase samples (day 56) without additional boosting. There was no significant difference in immunogenicity of crude versus pure OSP-core conjugate, although vaccination with crude OSP-core conjugate resulted in more prominent day 56 immune responses than immunization with conjugate from synthetic hexasaccharide lacking core (*P* < 0.05 [Fig. 3A]). There was also a trend toward more prominent immune responses following vaccination with conjugate containing tetrasaccharide versus hexasaccharide (*P* = 0.09). To assess whether these results correlated with total mass of saccharide used in vaccination, we also assessed immunogenicity following vaccination equalized by mass of “active” sugar (not including core oligosaccharide or any linkers) (Fig. 3B), as well as equalized by total moles of saccharide (Fig. 3C). Due to limited availability of reagents, for these analyses we assessed only conjugates containing native, pure OSP-core (conjugate 3b), synthetic OSP (conjugate 4b), and tetrasaccharide (conjugate 13b). Immune responses to native OSP-core and tetrasaccharide were comparable and significantly more prominent than responses to conjugate containing synthetic OSP (hexasaccharide lacking core; *P* ≤ 0.05 [Fig. 3B and C]). We performed similar analyses by judging immune responses not against LPS but against native, pure OSP-core (Fig. 4A), synthetic OSP (see Fig. S1 in the supplemental material), and synthetic tetrasaccharide (Fig. S2). Regarding OSP responses following vaccination with total saccharide mass-equalized vaccine, once again, we found that immune responses following vaccination of mice with conjugates containing crude or pure native OSP-core were comparable and were significantly higher than those induced by vaccination with hexasaccharide conjugate (*P* < 0.05 [Fig. 4A]). We also found that tetrasaccharide was more immunogenic than hexasaccharide in inducing OSP responses (*P* < 0.05 [Fig. 4A]). These relationships were still evident when we equalized for mass of active sugar or by mole of sugar per vaccination (Fig. 4B and C). Immune responses were more prominent when assessed against homologous antigen (Fig. S1 and S2).

10.1128/mSphere.00114-21.2 FIG S1 Synthetic O139 OSP (hexasaccharide, preparation 4):BSA-specific serum anti-O139 IgG responses in mice intramuscularly immunized with different fragments of OSP conjugated with rTTHc and unconjugated native pure OSP (preparation 3) equalized to total micrograms of saccharide (A), native OSP-core (pure):rTTHc (conjugate 3b), synthetic OSP:rTTHc (conjugate 4b), and synthetic tetrasaccharide:rTTHc (conjugate 13b) equalized to mass of active sugar component in micrograms (lacking core and linker) (B), and conjugates 3b, 4b, and 13b equalized to moles of saccharide (C). Means and standard errors of the means are reported for each group. *, statistically significant difference (*P* < 0.05) from baseline (day 0) titer; **, *P* ≤ 0.01. Download FIG S1, TIF file, 1.2 MB. Copyright © 2021 Kamruzzaman et al. 2021 Kamruzzaman et al. https://creativecommons.org/licenses/by/4.0/ This content is distributed under the terms of the Creative Commons Attribution 4.0 International license.

10.1128/mSphere.00114-21.3 FIG S2 Synthetic tetrasaccharide (preparation 13):BSA-specific serum anti-O139 IgG responses in mice intramuscularly immunized with different fragments of OSP conjugated with rTTHc and unconjugated native pure OSP (preparation 3) equalized to total micrograms of saccharide (A), native OSP-core (pure):rTTHc (conjugate 3b), synthetic OSP:rTTHc (conjugate 4b) and synthetic tetrasaccharide:rTTHc (conjugate 13b) equalized to the active sugar component in micrograms (lacking core and linker) (B), and conjugates 3b, 4b, and 13b equalized to moles of saccharide (C). Means and standard errors of the means are reported for each group. *, statistically significant difference (*P* < 0.05) from baseline (day 0) titer; **, *P* ≤ 0.01. Download FIG S2, TIF file, 1.1 MB. Copyright © 2021 Kamruzzaman et al. 2021 Kamruzzaman et al. https://creativecommons.org/licenses/by/4.0/ This content is distributed under the terms of the Creative Commons Attribution 4.0 International license.

### Vibriocidal assays.

**FIG 5:**
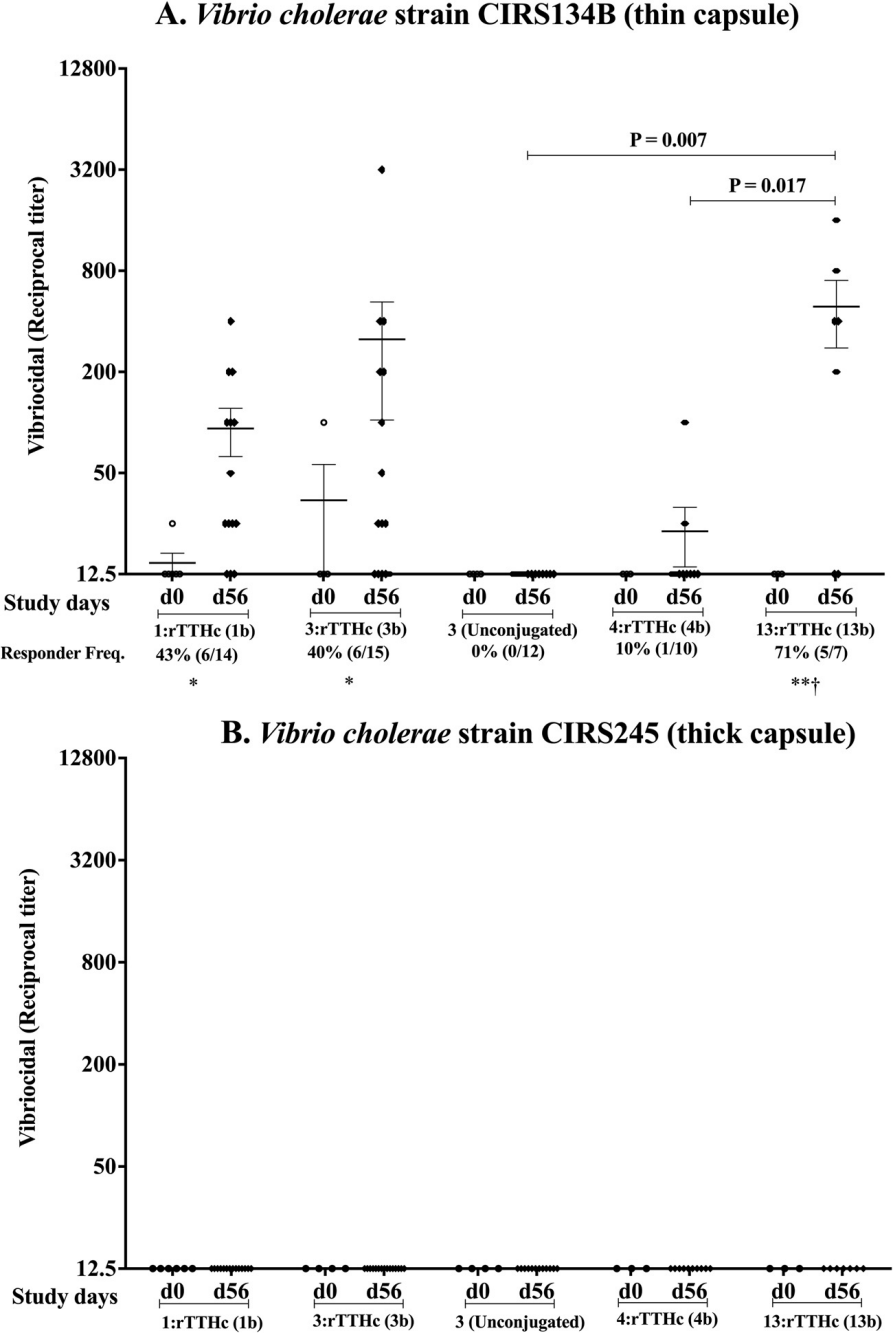
Vibriocidal antibody responses on day 56 in mice intramuscularly immunized with native OSPc:rTTHc (crude, conjugate 1b), native OSPc:rTTHc (pure, conjugate 3b), native pure OSPc unconjugated (preparation 3), synthetic OSP:rTTHc (conjugate 4b), or synthetic tetrasaccharide:rTTHc (conjugate 13b) against O139 strain CIRS134B (thinly encapsulated) (A) or against O139 strain CIRS245 (thickly encapsulated) (B). Responder frequency reflects a ≥4-fold increase in titer over the baseline (day 0) response. *, *P* ≤ 0.05 for increase (significant) in responder frequency compared to cohort receiving unconjugated vaccine; **, *P* ≤ 0.01 for increase (significant) in responder frequency compared to cohort receiving unconjugated vaccine; †, *P* ≤ 0.01 for increase (significant) in responder frequency compared to cohort receiving hexasaccharide vaccine.

To assess antibody functionality, we assessed vibriocidal antibody titers in serum of vaccinated mice. When assessing with a thinly encapsulated V. cholerae O139 strain (CIRS134B), we were unable to detect induction of vibriocidal responses in mice immunized with unconjugated native pure OSP-core lacking protein carrier (preparation 3 [Fig. 5A]). In comparison, mice immunized with conjugate vaccine containing crude or pure OSP-core developed significant vibriocidal responses (responder frequency increase, *P* < 0.05), and these responses were equivalent. Similar vibriocidal responses were induced by vaccination with tetrasaccharide conjugate 13b, with less prominent responses induced by vaccination with hexasaccharide conjugate 4b (*P* < 0.05). When the vibriocidal assay was repeated with the same serum samples but using thickly encapsulated strain of V. cholerae O139 CIRS245, we were unable to discern any vibriocidal activity (Fig. 5B).

### Protection in mouse neonatal challenge assay.

Serum from mice vaccinated with conjugate vaccines containing OSP-core or synthetic tetrasaccharide protected mice from challenge with virulent thinly encapsulated V. cholerae strain CIRS134B (37% survival in control mice challenged with virulent V. cholerae strain CIRS134B versus 83% survival in the presence of sera from mice previously vaccinated with pure OSPc:rTTHc [conjugate 3b; *P* = 0.008] versus 94% survival in the presence of sera of mice previously vaccinated with tetrasaccharide:rTTHc [conjugate 13b; *P* = 0.001] [Table 2]). We were, however, unable to discern protection from challenge using sera from mice previously vaccinated with conjugate 4b (59% survival; *P* = nonsignificant [NS]). When the assay included challenge with thickly encapsulated virulent V. cholerae O139 strain CIRS245 as opposed to thinly encapsulated strain CIRS134B, we were unable to discern protection using sera from any vaccine cohort, although serum from mice vaccinated with a conjugate from the synthetic tetrasaccharide approached statistical significance (44% survival with vaccine samples versus 16% for mice that were not vaccinated; conjugate 13b; *P* = 0.07).

**TABLE 2:** Survival at 36 h in mice challenged with virulent V. cholerae O139

Challenge strain	Vaccine cohort	*n*	% survival at 36 h	Vaccine efficacy (%)	*P* value^*a*^
V. cholerae O139-CIRS134B (thin capsule)		16	37.5		
	4:rTTHc (4b)	17	58.8	34	0.399
	13:rTTHc (13b)	17	94.1	91	0.0013^*b*^
	3:rTTHc (3b)	18	83.3	73	0.008^*c*^
V. cholerae O139-CIRS245 (thick capsule)		12	16.6		
	4:rTTHc (4b)	14	42.8	31	0.2
	13:rTTHc (13b)	9	44.4	33	0.07
	3:rTTHc (3b)	11	9.09	11	0.85

a Log rank comparison.

b Survival at 36 h by Fisher exact test, 0.0006.

c Survival at 36 h by Fisher exact test, 0.0061.

## DISCUSSION

V. cholerae O139 emerged as a cause of cholera in the early 1990s, rapidly spreading through 11 Asian counties before all but disappearing as a clinically important cause of cholera by 2005 (6, 21). V. cholerae O139 is still isolated from individuals in areas where cholera was previously endemic, but the organism has not led to explosive outbreaks for over two decades (6). The reasons for this are not clear. It is possible that V. cholerae O139 may have a survival disadvantage compared to V. cholerae O1 in the aquatic environmental reservoir where V. cholerae largely resides or that the presence of capsule may somehow affect human-to-human spread. When ingested by humans, both V. cholerae O139 and O1 can cause severe watery diarrhea, and the clinical presentations of the two types of cholera are indistinguishable (1, 3). V. cholerae O139 is highly similar to O1 and is thought to have evolved from a V. cholerae O1 El Tor strain (22).

The two serogroups have very high homology, including for cholera toxin. The primary difference between the two serogroups is in the *rfb* genes encoding the O-specific polysaccharide and the presence of a capsule in V. cholerae O139 that is absent from V. cholerae O1 (16, 23, 24). The OSP of V. cholerae O1 is a repetitive structure containing approximately 10 to 20 units of (1→2)-α-linked (4-*N*-3-deoxy-l-*glycero*-tetronyl)-perosamine, with or without a methyl group at *O*-2 on the terminal perosamine, distinguishing the Ogawa and Inaba serotypes, respectively. In comparison, the OSP of V. cholerae O139 is a single hexasaccharide whose structure was identified more than two decades ago (12, 13, 18). The fact that the latter OSP is a relatively simple molecule (a hexasaccharide) amenable to chemical synthesis and is not a polymer of an oligosaccharide repeating unit constitutes a unique situation among bacterial O-specific polysaccharides. Complex structure and polymolecularity make such substances unattached to the core virtually inaccessible in pure form either by regulated chemical synthesis or by isolation from bacteria. Availability of specimens of synthetic OSP of Vibrio cholerae O139, its fragments (25–30), and homogeneous native OSP-core (18) provided us with an unprecedented opportunity to subject these LPS-related antigens to the immunological studies presented here.

The OSP of both V. cholerae O1 and O139 serogroups is attached to lipid A via a core oligosaccharide that is the same in both V. cholerae O1 and O139. V. cholerae O139 also has a capsule, which is comprised of a polymer of O139 OSP not directly linked to lipid A and the bacterium. The thickness of the capsule of V. cholerae O139 varies by strain (21). As is the case with other encapsulated bacteria, it is hypothesized that the presence of capsule provides a survival advantage within infected hosts by blocking the ability of antibodies to bind to the bacterial cell surface and prevent or clear infection (31, 32). Importantly, despite the fact that V. cholerae O1 and O139 are highly homologous at the genomic level and produce essentially identical cholera toxin molecules, protection against cholera is serogroup specific, with serogroup specificity being defined by the OSP moiety (10). Previous infection with V. cholerae O139 provides protection against subsequent disease in human volunteer rechallenge models (33, 34). Protection of humans against O139 cholera is also afforded by previous ingestion of live attenuated strains of V. cholerae O139 lacking cholera holotoxin (33–35). Previous infection with V. cholerae O139, however, does not provide protection against infection with V. cholerae O1, and vice versa (10, 11). It is possible that V. cholerae O139 could reemerge as a cause of globally significant cholera or that other serogroups of V. cholerae with pandemic potential could emerge. For these reasons, we undertook this study to contribute to a better understanding of the immunoreactivity and immunogenicity of the various carbohydrate epitopes that comprise the OSP of V. cholerae O139 and related saccharides.

The OSP of V. cholerae O139 is a hexasaccharide containing *N*-acetyl-d-quinovosamine (QuiNAc), d-galacturonic acid (GalA), *N*-acetyl-d-glucosamine (GlcNAc), d-galactose (Gal) and two colitose (Col) residues. It contains two negatively charged groups, including a carboxyl group of d-galacturonic acid and a cyclic phosphate bound to *O*-4 and *O*-6 of the d-galactose (14, 15, 36). The capsule of V. cholerae O139 contains a flexible complex, branched polymer of this hexasaccharide (14, 15, 36). In our current analysis, we found marked reduction of immunoreactivity of convalescent-phase plasma when either one or both colitose residues and/or the cyclic phosphate were absent in O139 OSP fragments. We also found that the upstream terminal tetrasaccharide fragment was particularly immunoreactive with convalescent-phase human plasma and that a vaccine made from this determinant was particularly protective in mice. Previous analysis has disclosed that the terminal tetrasaccharide of V. cholerae O139 forms a very compact epitope with a relatively rigid conformation (37). Our observation of higher immunoreactivity of the tetrasaccharide than of both native OSP-core and synthetic OSP (full hexasaccharide) is consistent with the recognition of the OSP terminus by a cavity antibody-antigen interaction, as opposed to a groove-type interaction. The former type of interaction has previously been identified for V. cholerae O1 OSP (38) as well as some other bacterial surface polysaccharide interactions (39–42). Cavity antigen-antibody-type interactions can accommodate up to five sugar residues, while groove-type interactions can accommodate up to eight sugar residues (42). Cavity-type interactions have higher binding and complementarity and result in higher affinity and avidity than groove-type interactions (43).

We analyzed immunoreactivity by antibody isotype. Polysaccharides are classically considered T-cell-independent antigens and usually induce IgM responses. During cholera caused by V. cholerae O1, there is an initial IgM response to the O1 OSP; however, there is also rapid induction of class-switched IgA and IgG responses to this antigen (44). It has been hypothesized that this may relate to OSP being immunologically processed in the copresence of cholera holotoxin, which beyond being an enterotoxin is also a potent immunoadjuvant (44). In our current analysis of immune responses to human infection with V. cholerae O139, we also saw similar rapid induction of IgA and IgG responses to the O139 OSP. Previous analyses of OSP-specific monoclonal antibodies in patients with O1 cholera disclosed affinity maturation more akin to those induced to protein antigens than typically to polysaccharide antigens (45). Our results suggest that similar affinity maturation targeting polysaccharides may occur during O139 cholera as well.

In comparison to these responses in humans surviving naturally acquired O139 cholera, we were able to detect low-level IgM responses only to O139 OSP-core (native) and not to any synthetic or related preparations of O139 saccharides, and we detected no IgA or IgG responses to any O139 antigen in recipients of WC-O1/O139. Interestingly, baseline immunoreactivities (as judged by immunoreactivity of prevaccination day 0 samples) to a number of the polysaccharides analyzed in our profiling were higher in the samples from Haitian vaccinees than from Bangladeshi patients. This may reflect previous exposure to various polysaccharide or related antigens in these different populations. Cholera caused by V. cholerae O139 has never been recognized in Haiti. At present, the only commercially available vaccine targeting V. cholerae O139 is WC-O1/O139, a killed oral bivalent vaccine that includes heat- and formalin-inactivated strains of V. cholerae O1 and formalin-inactivated V. cholerae O139 strain 4260B (9). The vaccine has been safely administered to millions of humans, but O139-specific immune responses, including vibriocidal responses following vaccination, are significantly lower than those targeting O1 (9). Since O139 cholera is not presently a cause of epidemic cholera, and since no vaccine challenge studies have been performed focusing on O139, the ability of this bivalent vaccine to provide protection against O139, and the duration of any such protection should it exist, is currently unknown. Our results suggest that O139 OSP-specific responses are not prominent following WC-O1/O139 use in immunologically naive humans. This underscores the urgency to proactively develop a potent vaccine for the disease caused by V. cholerae O139.

Based on our immunoprofiling of O139 OSP-core and various fragments (Table 1), we further analyzed the ability of a subset of these antigens to mediate protection against cholera. Since direct evaluation in humans was not possible, we synthesized a number of conjugate vaccines with well-defined O139 cholera epitopes and used a mouse experimental challenge model. A number of cholera conjugate vaccines have been developed, including against V. cholerae O139 (36, 46–49). These previous vaccines were based on O139 capsule and derivatized LPS. They provided protection against V. cholerae challenge in animals (47, 48). In our current study, we used purified native OSP-core and well-defined synthetic oligosaccharide fragments thereof to further analyze these responses. We used a simple squaric acid-based conjugation technology (50–52) to attach these antigens in a sunburst non-cross-linked fashion, mimicking their display in nature, using essentially a technology we have used to develop a conjugate vaccine against V. cholerae O1 (19, 53–55). Similar to our observation that the terminal tetrasaccharide of V. cholerae O139 OSP was highly immunoreactive when analyzed with convalescent-phase human plasma, we also found conjugates prepared from it to be highly immunogenic in mice following vaccination. Indeed, it was more immunogenic than the conjugate made from complete synthetic OSP (hexasaccharide) and induced protection in the mouse model, while vaccination with the latter did not. Importantly, vaccination with native OSP-core conjugate was also more immunogenic than synthetic OSP hexasaccharide and provided better protection against challenge than did vaccination with the synthetic OSP lacking core. This could suggest that core-specific antibodies themselves might contribute to protection against O139 cholera. However, the fact that the cores of V. cholerae O139 and O1 are identical, but that there is no evident cross protection of O1 versus O139 cholera, suggests that even if core-specific antibodies do contribute some degree of protection, they are not clinically highly significant.

A plausible explanation for higher immunogenicity and protective capacity of the conjugate made from tetrasaccharide (conjugate 13) than of the one made from the complete synthetic OSP lacking the core (conjugate 4) could be that the absence of the downstream GalA-(1→4)-QuiNAc sequence in conjugate 13 allows the tetrasaccharide to be presented to immune cells in a more immunogenically relevant manner, e.g., allow it to attain a more favorable conformation, better fitting the subsite in the antibody binding area. Similar results have previously been reported in an analysis of immune responses to oligosaccharides related to the OSP of Shigella dysenteriae type 1 and their binding to a cavity-type monoclonal antibody (56). Nuclear magnetic resonance (NMR) analysis showed that the presence of sugar residues proximal to the immunodominant determinant affected conformation of the latter oligosaccharide, which, in turn, affected the antigen-antibody interaction.

In our analysis, the vibriocidal response and protection assays were strain dependent. Using the same convalescent-phase serum samples from vaccinated mice, we were unable to detect vibriocidal responses or protection when we used a thickly encapsulated strain of V. cholerae O139 but were able to show these when using a thinly encapsulated strain. The vibriocidal assay rests on binding of complement to the Fc fragment of bacterial surface-bound antibody that leads to generation of a terminal complement-based pore-forming membrane-attack complex (MAC) that inserts into the bacterial lipid membrane, resulting in lysis. The presence of a thick capsule could be assumed to lessen the likelihood that the MAC would be able to form or insert into the bacterial lipid membrane. The ability to assess vibriocidal responses against V. cholerae O139 is well known to be more variable than the ability to assess vibriocidal responses against V. cholerae O1 (17). Strain, capsule thickness, growth media, inoculum size, and concentration of complement have all been noted to play a role in giving rise to the ability of anti-O139 antibodies to lyse V. cholerae O139 (17).

Interestingly, in infection and rechallenge models of V. cholerae O139, and in vaccine challenge volunteer studies, the absence of demonstrable vibriocidal responses following first infection or vaccination and prior to challenge/rechallenge has sometimes not predicted protection against subsequent experimental infection (33–35, 57). A growing body of evidence also suggests that the vibriocidal activity is at best an imperfect predictor of protection against cholera caused by V. cholerae O1 and is optimally thought of as a surrogate marker of protection that largely reflects OSP-specific responses (58, 59). These observations underscore the difficulties inherent in using vibriocidal functional activity to predict protection. Despite this, in our current study, we were able to detect protection only using sera that also disclosed vibriocidal activity to a thinly encapsulated O139 strain, underscoring at least a surrogate relationship between this response and protection.

Our study has a number of limitations. It did not judge the ability of O139 polysaccharide-specific immune responses to mediate protection against O139 cholera in humans. Our human samples following cholera were collected relatively early in convalescence, and the duration of O139-specific immune responses that we detected is therefore uncertain. Our human samples were also limited, prompting us to use a pooled-sample analysis as opposed to assessing individual responses in infected humans. Our analysis also compared immune responses across Bangladeshi and Haitian populations, albeit matched by age, sex, and blood group. Our analysis also did not include assessment of mucosal or memory immune responses targeting V. cholerae O139. Despite these limitations, our analysis has defined O139-specific polysaccharide-specific immune responses following naturally acquired infection and vaccination. Our results suggest that protective antibodies described here are of the cavity type and that the upstream terminal tetrasaccharide of the OSP (itself a hexasaccharide) is the primary antigenic determinant. Our results also define two distinct epitopic regions as putative targets for antibody recognition: the two colitose residues and the cyclic 4,6-*O*-phosphate. If either of these structural elements is absent, the immunoreactivity of human plasma from patients who recover from O139 infection is significantly reduced. Our results also demonstrate that core oligosaccharide can contribute to immunogenicity, but its presence is not essential to induction of immune responses to O139 OSP. Our data show that relatively crude preparations of native OSP-core are as immunogenic and immunoreactive as the highly purified material and suggest that the crude native O139 OSP-core (18) or the immunodominant terminal tetrasaccharide (28) would be particularly attractive as candidates for antigenic components of a vaccine against V. cholerae O139. As cholera caused by V. cholerae O1 decreases with rollout and endorsement of vaccines effective against O1 cholera, development of a vaccine effective against V. cholerae O139 may be prudent (6).

## MATERIALS AND METHODS

### Ethics statement.

Our use of animals met all institutional and governmental requirements, guidelines, and policies. The Massachusetts General Hospital Subcommittee on Research Animal Care (SRAC) approved this work. This work adheres to the USDA Animal Welfare Act, PHS Policy on Humane Care and Use of Laboratory Animals, and the *Guide for the Care and Use of Laboratory Animals* (60). Plasma samples from humans recovering from culture-confirmed cholera caused by V. cholerae O139 and lacking other pathogens were collected from patients at the International Centre for Diarrhoeal Disease Research in Dhaka, Bangladesh (ICDDR,B). Plasma samples separated from blood were also collected as part of a research protocol approved by Zanmi Lasante Institutional Review Board (ZL0006303), from individuals who received the WHO-prequalified and -endorsed bivalent (WC-O1/O139) oral killed cholera vaccine, Shanchol (Sanofi-Shanta Biotech, India), in Haiti. This study was also approved by the Ethical Review and Research Review Committees of the ICDDR,B and the MassGeneral Brigham (MGB) Institutional Review Board, Boston, MA.

### Bacterial strains and media.

We prepared native LPS and OSP-core of V. cholerae O139 from strain CIRS245 as previously described (18). We used V. cholerae O139 strain CIRS245 (containing a thicker capsule) and strain CIRS134B (containing a thinner capsule) (21) in vibriocidal assays and in our mouse challenge assay. Strains were grown in Luria-Bertani (LB) broth.

### Production of native O139 OSP-core and synthetic oligosaccharides.

We generated three preparations of native V. cholerae O139 OSP-core fragment as previously described (18): (i) an initial preparation was generated by delipidation of LPS followed by purification by Bio-Gel P30 size exclusion chromatography (SEC), to afford material we termed “crude” O139 OSPc (preparation 1 [Table 1]); (ii) the crude preparation underwent additional purification, to substantial but not complete purity, by semipreparative high-performance liquid chromatography (HPLC), to obtain an “intermediate” preparation (preparation 2); and (iii) the intermediate preparation underwent further purification by analytical HPLC to obtain “pure” OSP-core (hexasaccharide-core, preparation 3), which was homogeneous by NMR spectroscopy, analytical HPLC, and electrospray ionization-mass spectrometry (ESI-MS), as previously described (18). We also synthesized the complete V. cholerae O139 OSP (hexasaccharide), as well as a series of derivatives and fragments of the OSP from disaccharide to hexasaccharide (conjugates 4 to 18 [Table 1]) (25–30). These include compounds lacking one or both colitose units or the phosphate residue or having a methyl galacturonate instead of a galacturonic acid residue. We conjugated these fragments, as well as our preparations of native OSP-core, to BSA or rTTHc using a squaric acid conjugation method as previously described (Table 1) (50–52).

### Assessing immunoreactivity of BSA conjugates using human plasma.

To assess immunoreactivity of O139 saccharides, we coated plates with 100 ng of antigen per well in 50 mM carbonate buffer (pH 9.6), as previously described (58, 61). We quantified polysaccharide-specific IgG, IgA, and IgM responses by enzyme-linked immunosorbent assay (ELISA) protocols as previously described (58), using acute-phase (day 2, following clinical stabilization and hydration) and convalescent-phase (day 7) plasma samples from 10 patients who recovered from O139 cholera in Dhaka, Bangladesh. We similarly assessed IgG, IgA, and IgM responses in recipients of WC-O1/O139 vaccine (Shanchol; Sanofi-Shantabiotech, India) in Haiti; vaccinees were matched by age group, sex, and blood group (O versus non-O) to naturally infected index patients (Table S1). WC-O1/O139 is administered as a two-dose oral vaccine (day 0 and day 14), and we assessed immune responses 7 days after the first and 7 days after the second dose of vaccine, comparing to that on day 0 (days 0, 7, and 21). We applied pooled 1:250 dilutions of serum in 0.1% BSA in phosphate-buffered saline (PBS)–0.05% Tween to coated plates, assessing immunoreactivity in triplicate. We detected antigen-specific antibodies using peroxidase-labeled goat anti-human IgG, IgM, or IgA antibody (Jackson ImmunoResearch, West Grove, PA; dilution, 1:5,000). After a 90-min incubation at 37°C, the plates were developed with a solution containing 0.55 mg/ml of 2,2*′*-*O*-azinobis(3-ethylbenzothiazoline-6-sulfonic acid) (ABTS; Sigma, St. Louis, MO) with 0.03% H_2_O_2_ (Sigma), and the changes in optical density (OD) at 405 nm were determined with a Vmax microplate kinetics reader (Molecular Devices Corp., Sunnyvale, CA). Plate readings were recorded for 5 min at 30-s intervals, and the maximum slope for an optical density change of 0.2 U was reported as milli-absorbance units (mAbs) per minute (58).

10.1128/mSphere.00114-21.4 TABLE S1 Blood group, sex and age group matching for Bangladeshi patients surviving naturally acquired O139 cholera and Haitian vaccine recipients of WC-O1/O139 (Shanchol; Sanofi-Shantabiotech, India). Download Table S1, DOCX file, 0.02 MB. Copyright © 2021 Kamruzzaman et al. 2021 Kamruzzaman et al. https://creativecommons.org/licenses/by/4.0/ This content is distributed under the terms of the Creative Commons Attribution 4.0 International license.

### Immunization of mice and collection of samples.

To assess immunogenicity of selected O139 saccharides, we used 8 cohorts (*n* = 7 to 15) of 3- to 5-week-old female Swiss Webster mice immunized intramuscularly with different preparations of O139 saccharides conjugated to rTTHc, as well as a control group vaccinated with unconjugated native pure OSP-core. We first equilibrated vaccine dose to total mass of saccharide (10 μg of sugar per mouse per vaccination). Mice were immunized on days 0, 21, and 42. We also assessed immunogenicity equilibrating to 10 μg of “active” sugar per mouse per vaccination. We defined active sugar as not including linker or core oligosaccharide. We also assessed immunogenicity equilibrating to moles, referencing from 10 μg of active sugar of OSPc:rTTHc. We collected blood samples via tail bleeds on days 0, 7, 21, 28, 42, 49, and 56 and processed, aliquoted, and stored samples as previously described (19, 53).

### Assessing immunogenicity in vaccinated mice.

We assessed immunogenicity in mouse serum collected from vaccinated mice, assessing responses to native O139 LPS, native OSP-core:BSA (conjugate 3a), synthetic OSP (hexasaccharide) absent core:BSA (conjugate 4a), and terminal tetrasaccharide fragment absent core:BSA (conjugate 13a) by using standard enzyme-linked immunosorbent assay (ELISA) protocols as previously described (19, 53). We coated plates with 100 ng of antigen per well to assess anti-OSP antibody responses and coated plates with 25 μg per well of LPS to assess anti-LPS antibody responses; we blocked, washed, and processed plates as previously described (19, 53). We used horseradish peroxidase-conjugated goat anti-mouse IgG antibody (dilution 1:1,000) as secondary antibody (Southern Biotech, Birmingham, AL). We normalized ELISA units (EU) by calculating the ratio of the optical density of test sample to a standard of pooled sera from control mice, as previously described (19, 53).

### Serum vibriocidal responses.

We assessed serum vibriocidal antibody titers in vaccinated mice, using V. cholerae O139 strain CIRS134B (thinly encapsulated) or CIRS245 (thickly encapsulated) in a microassay as previously described, with modification (53, 62, 63). We heat inactivated mouse sera for 1 h at 56°C. Heat-inactivated sera from mice were serially diluted 2-fold in 0.15 M saline, the dilutions ranging from 1:25 to 1:25,600, from which 50-μl volumes were added to the wells of sterile 96-well tissue culture plates already containing 50 μl of V. cholerae O139 strain CIRS134B or CIRS245 (OD, 0.1) in 0.15 M saline and 22% guinea pig complement (EMD Biosciences, San Diego, CA). We then incubated the plates for 1 h at 37°C and then added 150 μl of brain heart infusion medium (Becton, Dickinson, Sparks, MD) to each well, incubating plates for approximately 2 h at 37°C without shaking until absorbance (OD at 600 nm [OD_600_]) for growth control wells was between 0.2 and 0.3. At the conclusion of the assay, the vibriocidal titer was considered the serum dilution that generated a 50% reduction in optical density compared to that in the wells containing no serum (21). We considered a responder as having at least a 4-fold increase of vibriocidal titer at day 56 compared with the baseline day 0 titer.

### Neonatal challenge experiments.

We assessed the ability of serum from vaccinated mice to protect against challenge with wild-type virulent V. cholerae O139 CIRS134B or CIRS245, as previously described (19, 53). In brief, 3- to 5-day-old unimmunized CD-1 suckling mice (*n* = ∼15 mice/cohort) were removed from dams 2 h prior to inoculation. The mice were orally inoculated with 10^9^ CFU of V. cholerae O139 strain CIRS134B (50% lethal dose [LD_50_], ∼10^7-8^ CFU) or CIRS245 (LD_50_, ∼10^7^ CFU) mixed with a 1:1 dilution of pooled convalescent-phase day 56 serum from mice previously immunized with conjugate vaccines. Neonates were maintained at 30°C after the oral challenge and monitored every 3 h up to 36 h, following which the survivors were euthanized.

### Statistical analysis and graphs.

The data from different groups were compared using Mann-Whitney U tests. In addition, intragroup comparisons between the baseline (day 0) and other time points were made with Wilcoxon signed-rank tests. Survival curves generated from the neonatal challenge study were compared and analyzed with Kaplan-Meier and log rank analysis. The analyses were two tailed, and the cutoff *P* value of <0.05 was considered statistically significant. All statistical analyses were performed using GraphPad Prism 8 (GraphPad Software, Inc.).

10.1128/mSphere.00114-21.1 TEXT S1 General protocol for conjugating V. cholerae O139 OSP and its derivatives/fragments to carrier proteins using squaric acid chemistry. Download Text S1, DOCX file, 1.4 MB. Copyright © 2021 Kamruzzaman et al. 2021 Kamruzzaman et al. https://creativecommons.org/licenses/by/4.0/ This content is distributed under the terms of the Creative Commons Attribution 4.0 International license.

## References

[1] Albert MJ, Ansaruzzaman M, Bardhan PK, Faruque ASG, Faruque SM, Islam MS, Mahalanabis D, Sack RB, Salam MA, Siddique AK, Yunus MD, Zaman K. 1993. Large epidemic of cholera-like disease in Bangladesh caused by *Vibrio cholerae* O139 synonym Bengal. Lancet 342:387–390. 8101899

[2] Faruque SM, Chowdhury N, Kamruzzaman M, Ahmad QS, Faruque ASG, Salam MA, Ramamurthy T, Nair GB, Weintraub A, Sack DA. 2003. Reemergence of epidemic *Vibrio cholerae* O139, Bangladesh. Emerg Infect Dis 9:1116–1122. doi:10.3201/eid0909.020443. 14519249 PMC3016788

[3] Ramamurthy T, Garg S, Sharma R, Bhattacharya SK, Balakrish Nair G, Shimada T, Takeda T, Karasawa T, Kurazano H, Pal A, Takeda Y. 1993. Emergence of novel strain of *Vibrio cholerae* with epidemic potential in southern and eastern India. Lancet 341:703–704. doi:10.1016/0140-6736(93)90480-5. 8095620

[4] Rashed SM, Iqbal A, Mannan SB, Islam T, Rashid MU, Johura FT, Watanabe H, Hasan NA, Huq A, Stine OC, Sack RB, Colwell RR, Alam A. 2013. *Vibrio cholerae* O1 El Tor and O139 Bengal strains carrying ctxBET, Bangladesh. Emerg Infect Dis 19:1713–1715. doi:10.3201/eid1910.130626. 24050113 PMC3810759

[5] Alam M, Hasan NA, Sadique A, Bhuiyan NA, Ahmed KU, Nusrin S, Nair GB, Siddique AK, Sack RB, Sack DA, Huq A, Colwell RR. 2006. Seasonal cholera caused by *Vibrio cholerae* serogroups O1 and O139 in the coastal aquatic environment of Bangladesh. Appl Environ Microbiol 72:4096–4104. doi:10.1128/AEM.00066-06. 16751520 PMC1489596

[6] Chowdhury F, Mather AE, Begum YA, Asaduzzaman M, Baby N, Sharmin S, Biswas R, Uddin MI, LaRocque RC, Harris JB, Calderwood SB, Ryan ET, Clemens JD, Thomson NR, Qadri F. 2015. *Vibrio cholerae* serogroup O139: isolation from cholera patients and asymptomatic household family members in Bangladesh between 2013 and 2014. PLoS Negl Trop Dis 9:e0004183. doi:10.1371/journal.pntd.0004183. 26562418 PMC4642977

[7] Saha A, Chowdhury MI, Khanam F, Bhuiyan MS, Chowdhury F, Khan AI, Khan IA, Clemens J, Ali M, Cravioto A, Qadri F. 2011. Safety and immunogenicity study of a killed bivalent (O1 and O139) whole-cell oral cholera vaccine Shanchol, in Bangladeshi adults and children as young as 1 year of age. Vaccine 29:8285–8292. doi:10.1016/j.vaccine.2011.08.108. 21907255

[8] Bhattacharya SK, Sur D, Ali M, Kanungo S, You YA, Manna B, Sah B, Niyogi SK, Park JK, Sarkar B, Puri MK, Kim DR, Deen JL, Holmgren J, Carbis R, Dhingra MS, Donner A, Nair GB, Lopez AL, Wierzba TF, Clemens JD. 2013. 5 year efficacy of a bivalent killed whole-cell oral cholera vaccine in Kolkata, India: a cluster-randomised, double-blind, placebo-controlled trial. Lancet Infect Dis 13:1050–1056. doi:10.1016/S1473-3099(13)70273-1. 24140390

[9] Kanungo S, Lopez AL, Ali M, Manna B, Kim DR, Mahapatra T, Holmgren J, Dhingra MS, Weirzba TF, Nair GB, Bhattacharya SK, Clemens JD, Sur D. 2014. Vibriocidal antibody responses to a bivalent killed whole-cell oral cholera vaccine in a phase III trial in Kolkata, India. PLoS One 9:e96499. doi:10.1371/journal.pone.0096499. 24800828 PMC4011749

[10] Ali M, Emch M, Park JK, Yunus M, Clemens J. 2011. Natural cholera infection-derived immunity in an endemic setting. J Infect Dis 204:912–918. doi:10.1093/infdis/jir416. 21849288 PMC3156915

[11] Qadri F, Wennerås C, Albert MJ, Hossain J, Mannoor K, Begum YA, Mohi G, Salam MA, Sack RB, Svennerholm AM. 1997. Comparison of immune responses in patients infected with *Vibrio cholerae* O139 and O1. Infect Immun 65:3571–3576. doi:10.1128/iai.65.9.3571-3576.1997. 9284121 PMC175508

[12] Cox AD, Brisson JR, Varma V, Perry MB. 1996. Structural analysis of the lipopolysaccharide from *Vibrio cholerae* O139. Carbohydr Res 290:43–58. doi:10.1016/0008-6215(96)00135-8. 8805781

[13] Cox AD, Perry MB. 1996. Structural analysis of the O-antigen-core region of the lipopolysaccharide from *Vibrio cholerae* O139. Carbohydr Res 290:59–65. doi:10.1016/0008-6215(96)00131-0. 8805782

[14] Knirel YA, Paredes L, Jansson PE, Weintraub A, Widmalm G, Albert MJ. 1995. Structure of the capsular polysaccharide of *Vibrio cholerae* O139 synonym Bengal containing D‐galactose 4,6‐cyclophosphate. Eur J Biochem 232:391–396. doi:10.1111/j.1432-1033.1995.391zz.x. 7556186

[15] Preston LM, Xu Q, Johnson JA, Joseph A, Maneval DR, Husain K, Reddy GP, Bush CA, Morris JG. 1995. Preliminary structure determination of the capsular polysaccharide of *Vibrio cholerae* O139 Bengal Al1837. J Bacteriol 177:835–838. doi:10.1128/jb.177.3.835-838.1995. 7836323 PMC176667

[16] Waldor MK, Colwell R, Mekalanos JJ. 1994. The *Vibrio cholerae* O139 serogroup antigen includes an O-antigen capsule and lipopolysaccharide virulence determinants. Proc Natl Acad Sci U S A 91:11388–11392. doi:10.1073/pnas.91.24.11388. 7972070 PMC45236

[17] Attridge SR, Qadri F, Albert MJ, Manning PA. 2000. Susceptibility of *Vibrio cholerae* O139 to antibody-dependent, complement-mediated bacteriolysis. Clin Diagn Lab Immunol 7:444–450. doi:10.1128/CDLI.7.3.444-450.2000. 10799459 PMC95892

[18] Xu P, Korcová J, Baráth P, Čížová A, Valáriková J, Qadri F, Kelly M, O’Connor RD, Ryan ET, Bystrický S, Kováč P. 2019. Isolation, purification, characterization and direct conjugation of the lipid A-free lipopolysaccharide of *Vibrio cholerae* O139. Chemistry 25:12946–12956. doi:10.1002/chem.201902263. 31306528 PMC6783332

[19] Sayeed MA, Bufano MK, Xu P, Eckhoff G, Charles RC, Alam MM, Sultana T, Rashu MR, Berger A, Escobedo GG, Mandlik A, Bhuiyan TR, Leung DT, LaRocque RC, Harris JB, Calderwood SB, Qadri F, Vann WF, Kováč P, Ryan ET. 2015. A cholera conjugate vaccine containing O-specific polysaccharide (OSP) of *V. cholerae* O1 Inaba and recombinant fragment of tetanus toxin heavy chain (OSP:rTTHc) induces serum, memory and lamina proprial responses against OSP and is protective in mice. PLoS Negl Trop Dis 9:e0003881. doi:10.1371/journal.pntd.0003881. 26154421 PMC4495926

[20] Ou L, Kong WP, Chuang GY, Ghosh M, Gulla K, O’Dell S, Varriale J, Barefoot N, Changela A, Chao CW, Cheng C, Druz A, Kong R, McKee K, Rawi R, Sarfo EK, Schön A, Shaddeau A, Tsybovsky Y, Verardi R, Wang S, Wanninger TG, Xu K, Yang GJ, Zhang B, Zhang Y, Zhou T, Amharref N, Barry C, Boonyaratanakornkit B, Carey E, Caringal R, Carlton K, Chalamalsetty N, Charlton A, Chaudhuri R, Chen M, Chen P, Cibelli N, Cooper JW, Dahodwala H, Fleischman M, Frederick JC, Fuller H, Gall J, Godfroy I, Gollapudi D, Gowetski D, Horwitz J, Hussain A, The VRC Production Program, et al. 2020. Preclinical development of a fusion peptide conjugate as an HIV vaccine immunogen. Sci Rep 10:3032. doi:10.1038/s41598-020-59711-y. 32080235 PMC7033230

[21] Qadri F, Svennerholm AM, Shamsuzzaman S, Bhuiyan TR, Harris JB, Ghosh AN, Nair GB, Weintraub A, Faruque SM, Ryan ET, Sack DA, Calderwood SB. 2005. Reduction in capsular content and enhanced bacterial susceptibility to serum killing of *Vibrio cholerae* O139 associated with the 2002 cholera epidemic in Bangladesh. Infect Immun 73:6577–6583. doi:10.1128/IAI.73.10.6577-6583.2005. 16177333 PMC1230989

[22] Jonson G, Osek J, Svennerholm AM, Holmgren J. 1996. Immune mechanisms and protective antigens of *Vibrio cholerae* serogroup O139 as a basis for vaccine development. Infect Immun 64:3778–3785. doi:10.1128/IAI.64.9.3778-3785.1996. 8751929 PMC174293

[23] Berche P, Berche C, Abachin E, Lelievre H, Vandepitte J, Dodin A, Fournier JM. 1994. The novel epidemic strain O139 is closely related to the pandemic strain O1 of *Vibrio cholerae*. J Infect Dis 170:701–704. doi:10.1093/infdis/170.3.701. 8077733

[24] Comstock LE, Johnson JA, Michalski JM, Morris JG, Kaper JB. 1996. Cloning and sequence of a region encoding a surface polysaccharide of *Vibrio cholerae* O139 and characterization of the insertion site in the chromosome of *Vibrio cholerae* O1. Mol Microbiol 19:815–826. doi:10.1046/j.1365-2958.1996.407928.x. 8820651

[25] Soliman SE, Kováč P. 2016. Total synthesis of the complete protective antigen of *Vibrio cholerae* O139. Angew Chem Int Ed Engl 55:12850–12853. doi:10.1002/anie.201606116. 27623688 PMC5165651

[26] Lu X, Pfister HB, Soliman SE, Kováč P. 2018. O-specific polysaccharide of *Vibrio cholerae* O139: improved synthesis and conjugation to BSA by squaric acid chemistry. Eur J Org Chem 2018:2944–2957. doi:10.1002/ejoc.201800429.

[27] Soliman SE, Kováč P. 2015. Stereoselective syntheses of the conjugation-ready, downstream disaccharide and phosphorylated upstream, branched trisaccharide fragments of the O-PS of *Vibrio cholerae* O139. J Org Chem 80:4851–4860. doi:10.1021/acs.joc.5b00562. 25928584

[28] Ruttens B, Saksena R, Kováč P. 2007. Synthesis of phosphorylated, conjugation-ready di-, tri- and tetrasaccharide fragments of the O-specific polysaccharide of *V. cholerae* O139. Eur J Org Chem 2007:4366–4375. doi:10.1002/ejoc.200700322.

[29] Lu X, Kováč P. 2016. Chemical synthesis of the galacturonic acid containing pentasaccharide antigen of the O-specific polysaccharide of *Vibrio cholerae* O139 and its five fragments. J Org Chem 81:6374–6394. doi:10.1021/acs.joc.6b01019. 27452084

[30] Ruttens B, Kováč P. 2006. Synthesis of a phosphorylated disaccharide fragment of the O-specific polysaccharide of *Vibrio cholerae* O139, functionalized for conjugation. Helv Chim Acta 89:320–332. doi:10.1002/hlca.200690036.

[31] Weintraub A, Widmalm G, Jansson PE, Jansson M, Hultenby K, Albert MJ. 1994. *Vibrio cholerae* O139 Bengal possesses a capsular polysaccharide which may confer increased virulence. Microb Pathog 16:235–241. doi:10.1006/mpat.1994.1024. 8090081

[32] Nesper J, Schild S, Lauriano CM, Kraiss A, Klose KE, Reidl J. 2002. Role of *Vibrio cholerae* O139 surface polysaccharides in intestinal colonization. Infect Immun 70:5990–5996. doi:10.1128/IAI.70.11.5990-5996.2002. 12379674 PMC130371

[33] Losonsky GA, Lim Y, Motamedi P, Comstock LE, Johnson JA, Morris JG, Tacket CO, Kaper JB, Levine MM. 1997. Vibriocidal antibody responses in North American volunteers exposed to wild-type or vaccine *Vibrio cholerae* O139: specificity and relevance to immunity. Clin Diagn Lab Immunol 4:264–269. doi:10.1128/cdli.4.3.264-269.1997. 9144361 PMC170516

[34] Morris JG, Losonsky GE, Johnson JA, Tacket CO, Nataro JP, Panigrahi P, Levin MM. 1995. Clinical and immunologic characteristics of *Vibrio cholerae* O139 Bengal infection in North American volunteers. J Infect Dis 171:903–908. doi:10.1093/infdis/171.4.903. 7706818

[35] Coster TS, Killeen KP, Waldor MK, Beattie DT, Spriggs DR, Kenner JR, Trofa A, Sadoff JC, Mekalanos JJ, Taylor DN. 1995. Safety, immunogenicity, and efficacy of live attenuated *Vibrio cholerae* O139 vaccine prototype. Lancet 345:949–952. doi:10.1016/S0140-6736(95)90698-3. 7715293

[36] Kossaczka Z, Szu SC. 2000. Evaluation of synthetic schemes to prepare immunogenic conjugates of *Vibrio cholerae* O139 capsular polysaccharide with chicken serum albumin. Glycoconj J 17:425–433. doi:10.1023/a:1007164216202. 11294508

[37] Gunawardena S, Fiore CR, Johnson JA, Bush CA. 1999. Conformation of a rigid tetrasaccharide epitope in the capsular polysaccharide of *Vibrio cholerae* O139. Biochemistry 38:12062–12071. doi:10.1021/bi9910272. 10508410

[38] Villeneuve S, Souchon H, Riottot MM, Mazié JC, Lei PS, Glaudemans CPJ, Kováč P, Fournier JM, Alzari PM. 2000. Crystal structure of an anti-carbohydrate antibody directed against *Vibrio cholerae* O1 in complex with antigen: molecular basis for serotype specificity. Proc Natl Acad Sci U S A 97:8433–8438. doi:10.1073/pnas.060022997. 10880560 PMC26965

[39] Rose DR, Przybylska M, To RJ, Kayden CS, Vorberg E, Young NM, Bundle DR, Oomen RP. 1993. Crystal structure to 2.45 Å resolution of a monoclonal Fab specific for the Brucella A cell wall polysaccharide antigen. Protein Sci 2:1106–1113. doi:10.1002/pro.5560020705. 8358294 PMC2142428

[40] Carlin NI, Bundle DR, Lindberg AA. 1987. Characterization of five *Shigella flexneri* variant Y-specific monoclonal antibodies using defined saccharides and glycoconjugate antigens. J Immunol 138:4419–4427. 2438343

[41] Vyas NK, Vyas MN, Chervenak MC, Johnson MA, Pinto BM, Bundle DR, Quiocho FA. 2002. Molecular recognition of oligosaccharide epitopes by a monoclonal Fab specific for *Shigella flexneri* Y lipopolysaccharide: X-ray structures and thermodynamics. Biochemistry 41:13575–13586. doi:10.1021/bi0261387. 12427018

[42] Roche MI, Lu Z, Hui JH, Sharon J. 2011. Characterization of monoclonal antibodies to terminal and internal O-antigen epitopes of *Francisella tularensis* lipopolysaccharide. Hybridoma (Larchmt) 30:19–28. doi:10.1089/hyb.2010.0083. 21466282 PMC3119334

[43] Cisar J, Kabat EA, Dorner MM, Liao J. 1975. Binding properties of immunoglobulin combining sites specific for terminal or nonterminal antigenic determinants in dextran. J Exp Med 142:435–459. doi:10.1084/jem.142.2.435. 49389 PMC2189905

[44] Hossain M, Islam K, Kelly M, Smith LMM, Charles RC, Weil AA, Bhuiyan TR, Kováč P, Xu P, Calderwood SB, Simon JK, Chen WH, Lock M, Lyon CE, Kirkpatrick BD, Cohen M, Levine MM, Gurwith M, Leung DT, Azman AS, Harris JB, Qadri F, Ryan ET. 2019. Immune responses to O-specific polysaccharide (OSP) in North American adults infected with *Vibrio cholerae* O1 Inaba. PLoS Negl Trop Dis 13:e0007874. doi:10.1371/journal.pntd.0007874. 31743334 PMC6863522

[45] Kauffman RC, Bhuiyan TR, Nakajima R, Mayo-Smith LM, Rashu R, Hoq MR, Chowdhury F, Khan AI, Rahman A, Bhaumik SK, Harris L, O’Neal JT, Trost JF, Alam NH, Jasinskas A, Dotsey E, Kelly M, Charles RC, Xu P, Kováč P, Calderwood SB, Ryan ET, Felgner PL, Qadri F, Wrammert J, Harris JB. 2016. Single-cell analysis of the plasmablast response to *Vibrio cholerae* demonstrates expansion of cross-reactive memory B cells. mBio 7:e02021-16. doi:10.1128/mBio.02021-16. 27999163 PMC5181778

[46] Kossaczka Z, Shiloach J, Johnson V, Taylor DN, Finkelstein RA, Robbins JB, Szu SC. 2000. *Vibrio cholerae* O139 conjugate vaccines: synthesis and immunogenicity of *V. cholera*e O139 capsular polysaccharide conjugates with recombinant diphtheria toxin mutant in mice. Infect Immun 68:5037–5043. doi:10.1128/IAI.68.9.5037-5043.2000. 10948122 PMC101731

[47] Johnson JA, Joseph A, Morris JG. 1995. Capsular polysaccharide-protein conjugate vaccines against *Vibrio cholera*e O139 Bengal. Bull Inst Pasteur 93:285–290. doi:10.1016/0020-2452(96)85763-0.

[48] Boutonnier A, Villeneuve S, Nato F, Dassy B, Fournier JM. 2001. Preparation, immunogenicity, and protective efficacy, in a murine model, of a conjugate vaccine composed of the polysaccharide moiety of the lipopolysaccharide of *Vibrio cholerae* O139 bound to tetanus toxoid. Infect Immun 69:3488–3493. doi:10.1128/IAI.69.5.3488-3493.2001. 11292781 PMC98317

[49] Fleischhackerová A, Farkaš P, Čížová A, Bystrický S. 2014. Preparation and immunogenicity of conjugate based on hydrazine-treated lipopolysaccharide antigen of *Vibrio cholerae* O139. Biosci Biotechnol Biochem 78:1817–1824. doi:10.1080/09168451.2014.942251. 25070088

[50] Kováč P, Xu P. 2017. Controlled and highly efficient preparation of carbohydrate-based vaccines: squaric acid chemistry is the way to go. Carbohydr Chem 42:83–115.

[51] Pfister HB, Lu X, Soliman SE, Kováč P. 2019. Conjugation of synthetic oligosaccharides to proteins by squaric acid chemistry. Methods Mol Biol 1954:77–88. doi:10.1007/978-1-4939-9154-9_7. 30864125

[52] Xu P, Kováč P. 2019. Direct conjugation of bacterial polysaccharides to proteins by squaric acid chemistry. Methods Mol Biol 1954:89–98. doi:10.1007/978-1-4939-9154-9_8. 30864126

[53] Alam MM, Bufano MK, Xu P, Kalsy A, Yu Y, Freeman YW, Sultana T, Rashu MR, Desai I, Eckhoff G, Leung DT, Charles RC, LaRocque RC, Harris JB, Clements JD, Calderwood SB, Qadri F, Vann WF, Kováč P, Ryan ET. 2014. Evaluation in mice of a conjugate vaccine for cholera made from *Vibrio cholerae* O1 (Ogawa) O-specific polysaccharide. PLoS Negl Trop Dis 8:e2683. doi:10.1371/journal.pntd.0002683. 24516685 PMC3916310

[54] Xu P, Alam MM, Kalsy A, Charles RC, Calderwood SB, Qadri F, Ryan ET, Kováč P. 2011. Simple, direct conjugation of bacterial O-SP-core antigens to proteins: development of cholera conjugate vaccines. Bioconjug Chem 22:2179–2185. doi:10.1021/bc2001984. 21899371 PMC3197769

[55] Xu P, Kelly M, Vann WF, Qadri F, Ryan ET, Kováč P. 2017. Conjugate vaccines from bacterial antigens by squaric acid chemistry: a closer look. Chembiochem 18:799–815. doi:10.1002/cbic.201600699. 28182850 PMC5664186

[56] Pavliak V, Nashed EM, Pozsgay V, Kovác P, Karpas A, Chu C, Schneerson R, Robbins JB, Glaudemans CP. 1993. Binding of the O-antigen of *Shigella dysenteriae* type 1 and 26 related synthetic fragments to a monoclonal IgM antibody. J Biol Chem 268:25797–25802. doi:10.1016/S0021-9258(19)74460-3. 7503987

[57] Tacket CO, Losonsky G, Nataro JP, Comstock L, Michalski J, Edelman R, Kaper JB, Levine MM. 1995. Initial clinical studies of CVD 112 *Vibrio cholerae* O139 live oral vaccine: safety and efficacy against experimental challenge. J Infect Dis 172:883–886. doi:10.1093/infdis/172.3.883. 7658089

[58] Akter A, Dash P, Aktar A, Jahan SR, Afrin S, Basher SR, Hakim A, Lisa AK, Chowdhury F, Khan AI, Xu P, Charles RC, Kelly M, Kováč P, Harris JB, Bhuiyan TR, Calderwood SB, Ryan ET, Qadri F. 2019. Induction of systemic, mucosal and memory antibody responses targeting *Vibrio cholerae* O1 O-specific polysaccharide (OSP) in adults following oral vaccination with an oral killed whole cell cholera vaccine in Bangladesh. PLoS Negl Trop Dis 13:e0007634. doi:10.1371/journal.pntd.0007634. 31369553 PMC6692040

[59] Leung DT, Rahman MA, Mohasin M, Riyadh MA, Patel SM, Alam MM, Chowdhury F, Khan AI, Kalivoda EJ, Aktar A, Bhuiyan MS, LaRocque RC, Harris JB, Calderwood SB, Qadri F, Ryan ET. 2011. Comparison of memory B cell, antibody-secreting cell, and plasma antibody responses in young children, older children, and adults with infection caused by *Vibrio cholerae* O1 El Tor Ogawa in Bangladesh. Clin Vaccine Immunol 18:1317–1325. doi:10.1128/CVI.05124-11. 21697337 PMC3147357

[60] National Research Council. 2011. Guide for the care and use of laboratory animals, 8th ed. National Academies Press, Washington, DC.

[61] Uddin T, Aktar A, Xu P, Johnson RA, Rahman MA, Leung DT, Afrin S, Akter A, Alam MM, Rahman A, Chowdhury F, Khan AI, Bhuiyan TR, Bufano MK, Rashu R, Yu Y, Wu-Freeman Y, Harris JB, LaRocque RC, Charles RC, Kováč P, Calderwood SB, Ryan ET, Qadri F. 2014. Immune responses to O-specific polysaccharide and lipopolysaccharide of *Vibrio cholerae* O1 Ogawa in adult Bangladeshi recipients of an oral killed cholera vaccine and comparison to responses in patients with cholera. Am J Trop Med Hyg 90:873–881. doi:10.4269/ajtmh.13-0498. 24686738 PMC4015581

[62] Ryan ET, Butterton JR, Zhang T, Baker MA, Stanley SL, Calderwood SB. 1997. Oral immunization with attenuated vaccine strains of *Vibrio cholerae* expressing a dodecapeptide repeat of the serine-rich *Entamoeba histolytica* protein fused to the cholera toxin B subunit induces systemic and mucosal antiamebic and anti-*V. cholerae* antibody responses in mice. Infect Immun 65:3118–3125. doi:10.1128/iai.65.8.3118-3125.1997. 9234763 PMC175440

[63] Qadri F, Mohi G, Hossain J, Azim T, Khan AM, Salam MA, Sack RB, Albert MJ, Svennerholm AM. 1995. Comparison of the vibriocidal antibody response in cholera due to *Vibrio cholerae* O139 Bengal with the response in cholera due to *Vibrio cholerae* O1. Clin Diagn Lab Immunol 2:685–688. doi:10.1128/cdli.2.6.685-688.1995. 8574829 PMC170220

