# Supplementary figures and images for: Defining Polysaccharide-Specific Antibody Targets against Vibrio cholerae O139 in Humans following O139 Cholera and following Vaccination with a Commercial Bivalent Oral Cholera Vaccine, and Evaluation of Conjugate Vaccines Targeting O139
Source: mSphere. 2021 Jul 7;6(4):e00114-21. doi: 10.1128/mSphere.00114-21 (PMC8386440; doi:10.1128/mSphere.00114-21)

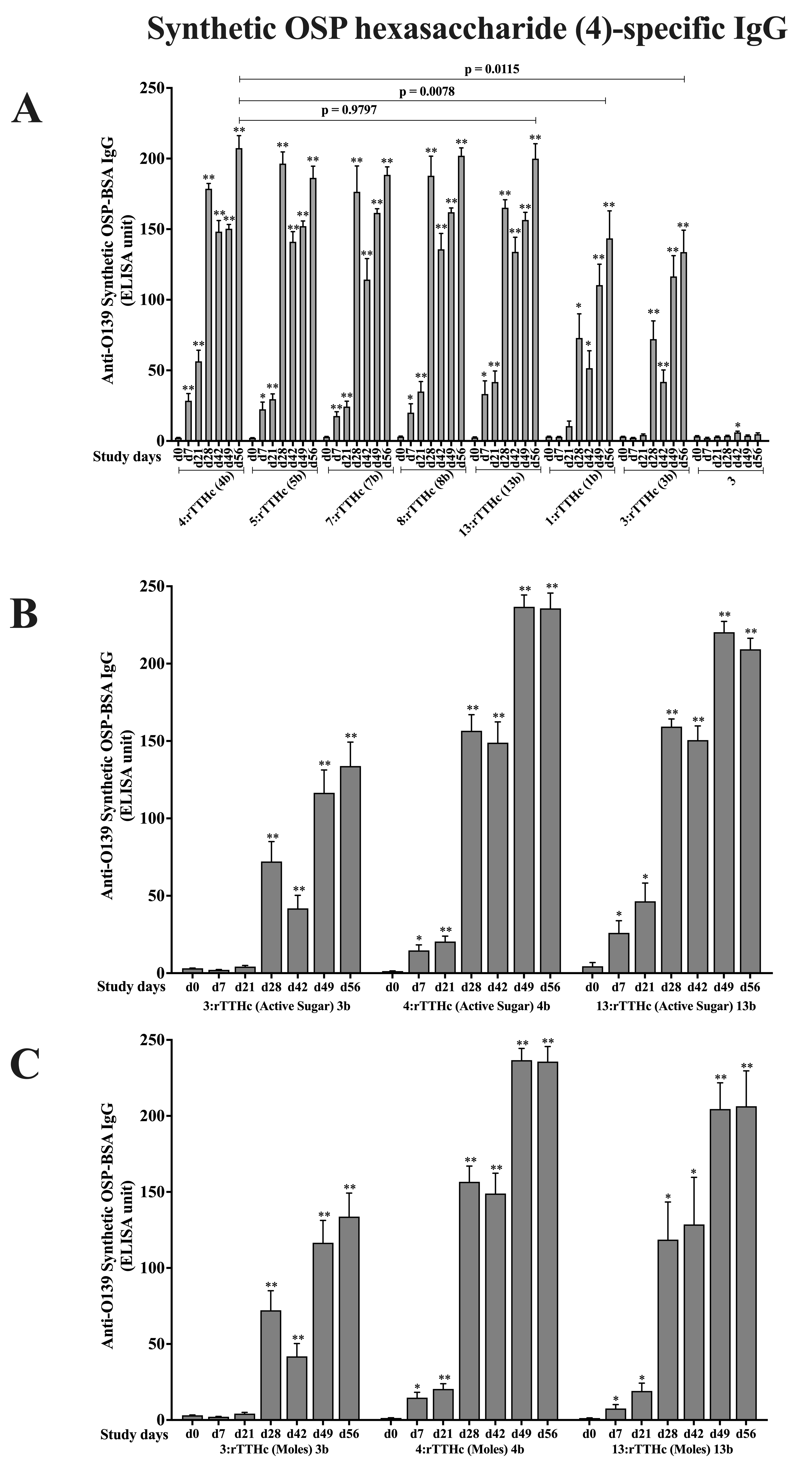

Supplement: FIG S1 [file msphere.00114-21-sf001.tif]

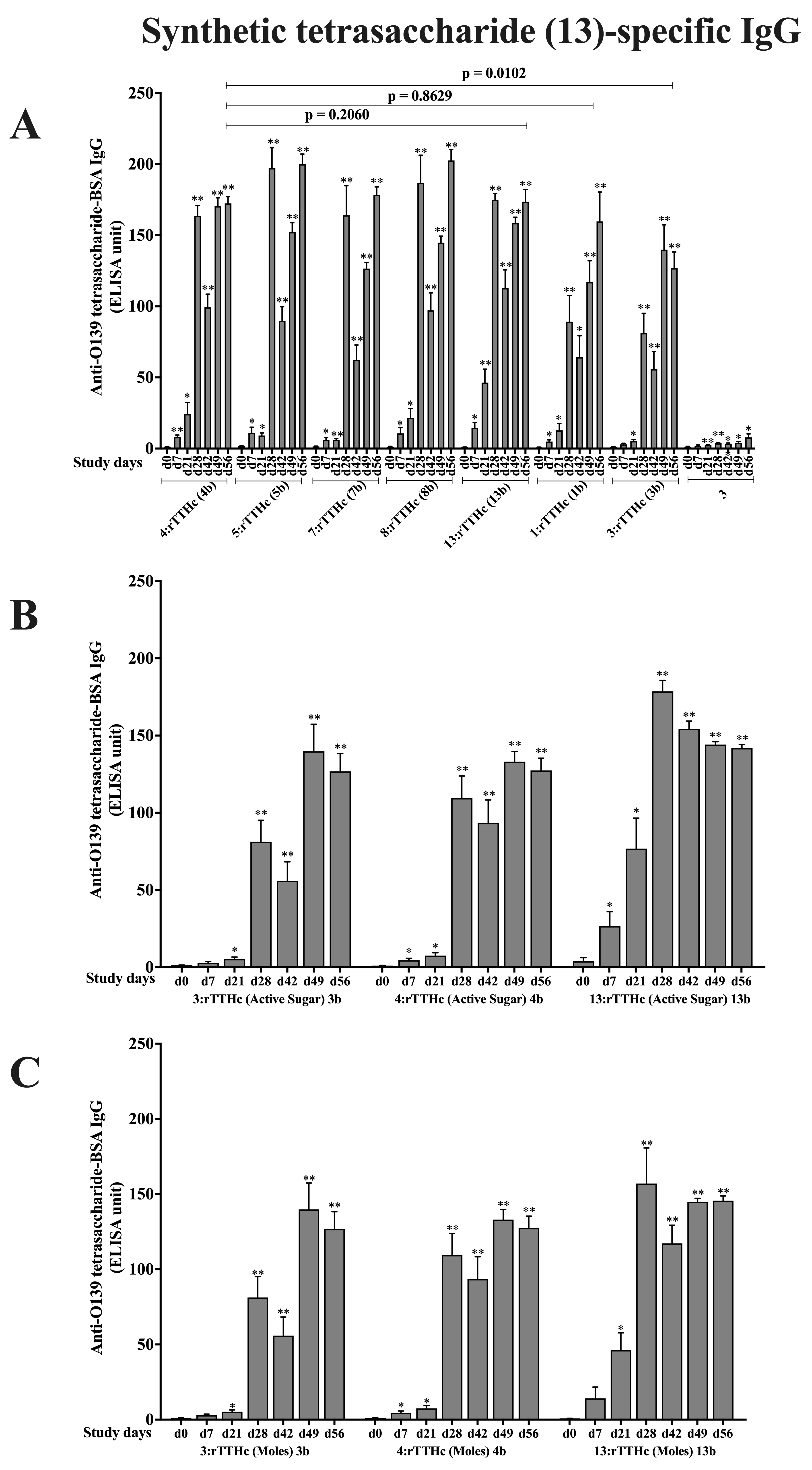

Supplement: FIG S2 [file msphere.00114-21-sf002.tif]
