# Supplementary material for: Defining Polysaccharide-Specific Antibody Targets against Vibrio cholerae O139 in Humans following O139 Cholera and following Vaccination with a Commercial Bivalent Oral Cholera Vaccine, and Evaluation of Conjugate Vaccines Targeting O139
Source: mSphere. 2021 Jul 7;6(4):e00114-21. doi: 10.1128/mSphere.00114-21 (PMC8386440; doi:10.1128/mSphere.00114-21)
Supplement: TABLE S1 [file msphere.00114-21-st001.docx]

**Table S1: Blood group, gender, and age group matching for Bangladeshi patients surviving naturally acquired O139-cholera, and Haitian vaccine recipients of WC-O1/O139 (Shanchol; Sanofi-Shantabiotech, India).**

| **Naturally acquired O139-cholera (Bangladesh)** | | |  | **Vaccine recipients of Bi-kOCV**  **(Haiti)** | | |
| --- | --- | --- | --- | --- | --- | --- |
| **Age (years)** | **Gender** | **Blood Group** |  | **Age (years)** | **Gender** | **Blood Group** |
| 7 | F | B |  | 7 | F | B |
| 18 | F | B |  | 14 | F | B |
| 21 | F | O |  | 21 | F | O |
| 21 | F | O |  | 22 | F | O |
| 31 | F | O |  | 31 | F | O |
| 35 | F | O |  | 35 | F | O |
| 41 | F | A |  | 40 | F | A |
| 60 | M | B |  | 39 | M | B |
| 60 | M | B |  | 60 | M | B |
| 65 | M | O |  | 52 | M | O |

* Primary matching was to age group (child, <10 years of age; adolescent 10-18 years of age; adult, >18 years of age), blood group (O versus non-O), and gender. Within age grouping, we then matched individuals by closest age in years with matching blood group and gender.
