# Supplementary material for: Defining Polysaccharide-Specific Antibody Targets against Vibrio cholerae O139 in Humans following O139 Cholera and following Vaccination with a Commercial Bivalent Oral Cholera Vaccine, and Evaluation of Conjugate Vaccines Targeting O139
Source: mSphere. 2021 Jul 7;6(4):e00114-21. doi: 10.1128/mSphere.00114-21 (PMC8386440; doi:10.1128/mSphere.00114-21)
Supplement: TEXT S1 [file msphere.00114-21-s0001.docx]

**Supplementary Methods**

**Method S1: General protocol for conjugating *V. cholerae* O139 OSP and its derivatives/fragments to carrier proteins using squaric acid chemistry.**

For detailed protocols about squaric acid conjugation, please refer to ref.S1-3. In brief, amine antigen (1 eq) was dissolved in pH 7 phosphate butter (0.5 M) to form a 4 mM solution followed by adding 3,4-Dimethoxy-3-cyclobutene-1,2-dione (1.5-10 eq. In the case of disaccharide **17**, 1.25 eq of 3,4-diethoxy-3-cyclobutene-1,2-dione was used). The clear solution formed was stirred at room temperature for 6-18 h until TLC showed disappearance of the starting material and formation of a faster moving spot. The reaction mixture was purified by size-exclusion chromatography using P-2 Bio-Gel (Bio-Rad, elute with water) or by HPLC (Agilent 1100 series with MWD. Column: XBridge BEH Amide 5μm, 19x250 mm, Waters). The fractions containing the methyl squarate derivative of the antigen were combined and freeze-dried to give a white solid product. For conjugation, the squarate intermediate obtained (6-10 eq) and carrier protein (BSA or rTT-Hc, 1 eq) were placed in a reaction vial and dissolved in 0.5 M pH 9 borate buffer to form a 4 mM solution with respect to the squarate derivative of antigen. The clear solution formed was stirred at room temperature for 3-5 days until the desired antigen to protein ratio was reached (checked by MALDI-TOF-MS or SELDI-TOF-MS). The reaction mixture was purified by ultrafiltration using Amicon Ultrafiltration devices (SigmaMillipore, 30k Da cut-off). The conjugate in the retentate, after freeze-drying, was obtained as a white fluffy solid.

The preparation of conjugates **1a**-**3a**^S4^, **4a**-**6a**^S5^ has been previously described.

**7a:**

Antigen **7** ^S6^ (5.28 mg, 5.21 μmol) was treated with 3,4-Dimethoxy-3-cyclobutene-1,2-dione (7.4 mg, 52.1 μmol) in pH 7 buffer for 15h to give squarate derivative (5.8 mg, 99%) as a white solid. HRMS-ESI: m/z [M+NH_4_]^+^ calcd for C_43_H_71_N_3_O_30_P: 1140.3860; found: 1140.3859. Methyl squarate derivative of **7** (0.22 mg, 0.196 μmol) was conjugated to BSA (2.17 mg, 0.0327 μmol) in pH 9 buffer to give 2.2 mg of white fluffy solid conjugate **7a** (92%). Estimated antigen to protein ratio: 6.0. SELDI-TOF-MS of **7a** is shown below:

**
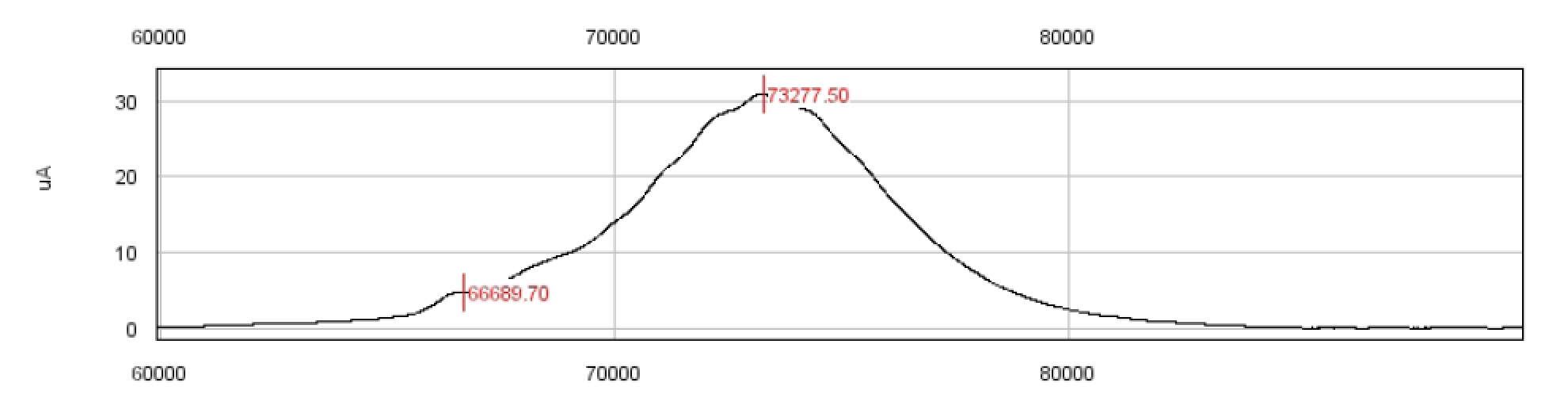
**

**8a:**

Antigen **8**^S6^ (5.22 mg, 5.08 μmol) was treated with 3,4-Dimethoxy-3-cyclobutene-1,2-dione (7.22 mg, 50.8 μmol) in pH 7 buffer for 15h to give squarate derivative (5.7 mg, 99%) as a white solid. HRMS-ESI: m/z [M+NH_4_]^+^ calcd for C_44_H_73_N_3_O_30_P: 1154.4016; found: 1154.4014. Methyl squarate derivative of **8** (0.27 mg, 0.261 μmol) was conjugated to BSA (2.9 mg, 0.0437 μmol) in pH 9 buffer to give 2.93 mg of white fluffy solid conjugate **8a** (94%). Estimated antigen to protein ratio: 4.6. SELDI-TOF-MS of **8a** is shown below:

**
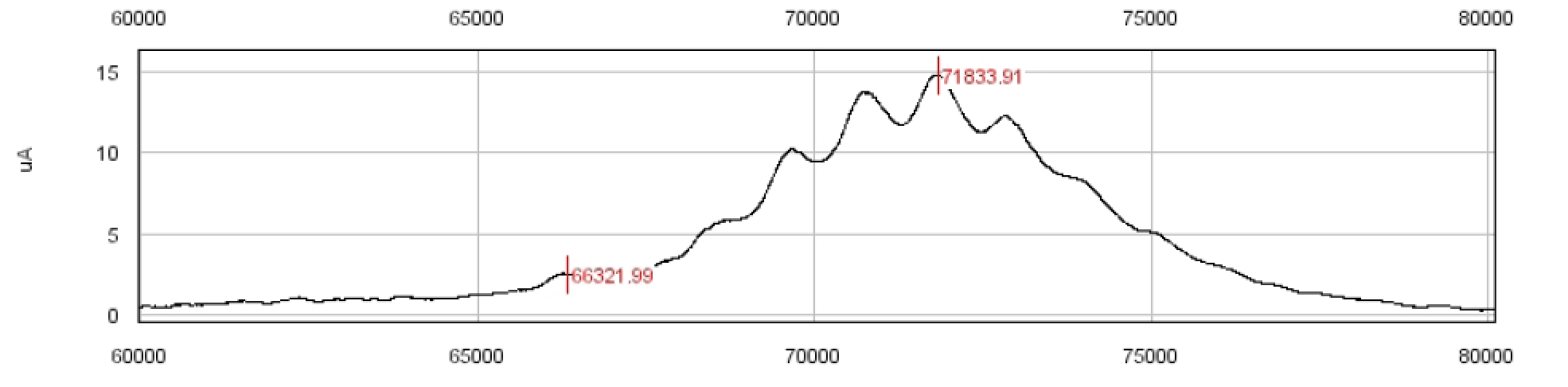
**

**9a:**

Antigen **9**^S6^ (5.0 mg, 5.26 μmol) was treated with 3,4-Dimethoxy-3-cyclobutene-1,2-dione (4.2 mg, 29.5 μmol) in pH 7 buffer for 16h to give squarate derivative (3.0 mg, 54%) as a white solid. HRMS-ESI: m/z [M+Na]^+^ calcd for C_43_H_68_N_2_O_28_Na: 1083.3856; found: 1083.3861. Methyl squarate derivative of **9** (0.26 mg, 0.245 μmol) was conjugated to BSA (2.7 mg, 0.0406 μmol) in pH 9 buffer to give 2.3 mg of white fluffy solid conjugate **9a** (79%). Estimated antigen to protein ratio: 5.1. SELDI-TOF-MS of **9a** is shown below:

**
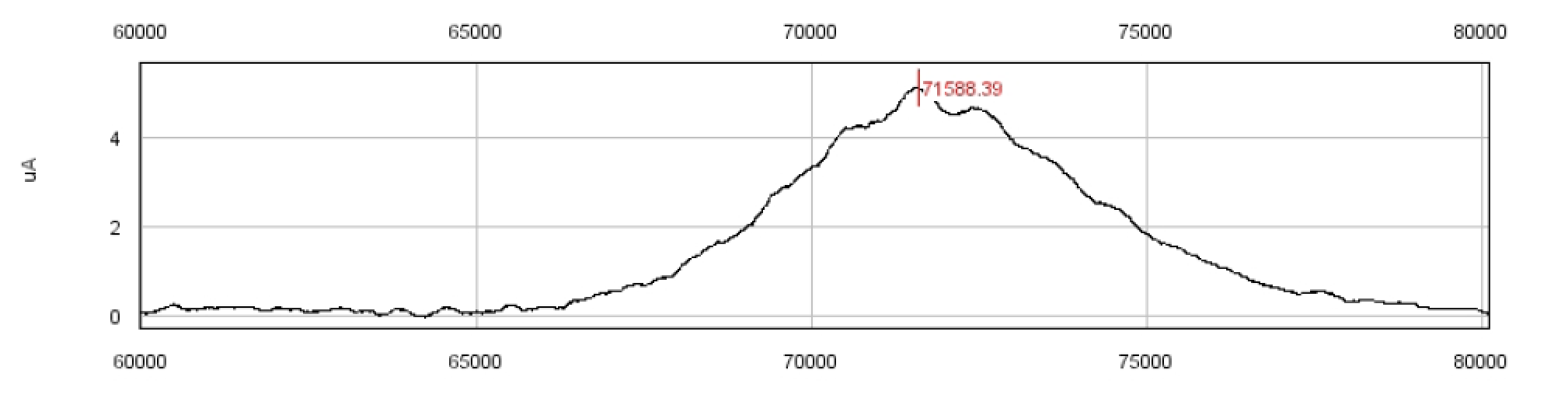
**

**10a:**

Antigen **10**^S6^ (5.8 mg, 6.6 μmol) was treated with 3,4-Dimethoxy-3-cyclobutene-1,2-dione (1.4 mg, 10 μmol) in pH 7 buffer for 16h to give squarate derivative (2.2 mg, 34%, purified by HPLC eluted with MeCN:MeOH:H_2_O/60:20:20) as a white solid. HRMS-ESI: m/z [M+Na]^+^ calcd for C_37_H_57_N_2_O_27_NaP: 1015.2784; found: 1015.2801. Methyl squarate derivative of **10** (0.36 mg, 0.363 μmol) was conjugated to BSA (4.0 mg, 0.0605 μmol) in pH 9 buffer to give 3.6 mg of white fluffy solid conjugate **10a** (83%). Estimated antigen to protein ratio: 4.9. SELDI-TOF-MS of **10a** is shown below (the signal at 66,608.26 Da is a signal of BSA added as internal standard into the SELDI sample):

**
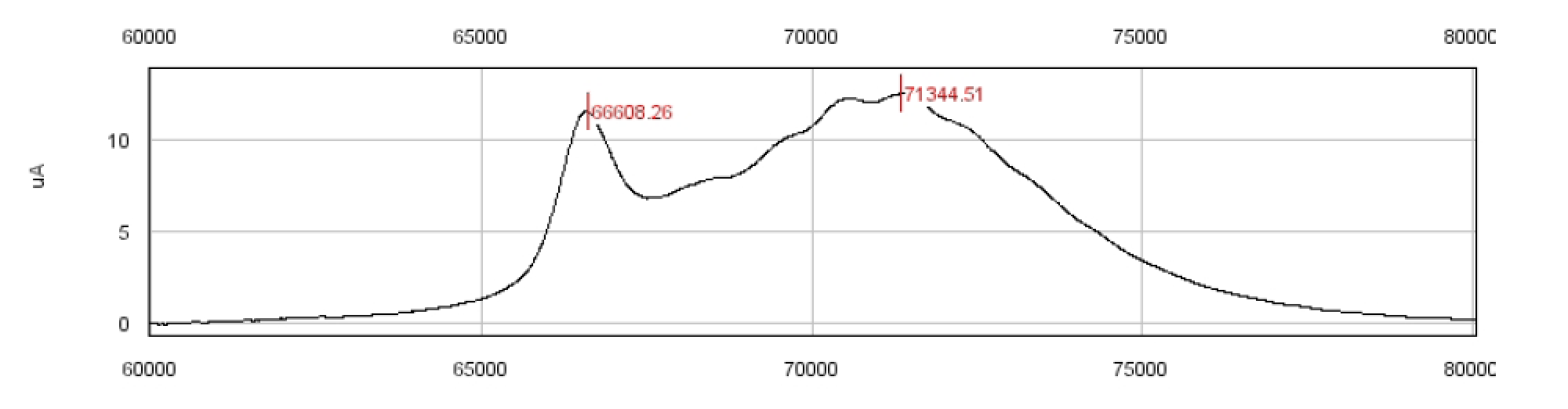
**

**11a:**

Antigen **11**^S6^ (5.8 mg, 6.6 μmol) was treated with 3,4-Dimethoxy-3-cyclobutene-1,2-dione (1.4 mg, 10 μmol) in pH 7 buffer for 16h to give squarate derivative (2.3 mg, 35%, purified by HPLC eluted with MeCN:MeOH:H_2_O/60:20:20) as a white solid. HRMS-ESI: m/z [M+Na]^+^ calcd for C_37_H_57_N_2_O_27_NaP: 1015.2784; found: 1015.2779. Methyl squarate derivative of **11** (0.33 mg, 0.332 μmol) was conjugated to BSA (3.67 mg, 0.055 μmol) in pH 9 buffer to give 2.8 mg of white fluffy solid conjugate **11a** (72%). Estimated antigen to protein ratio: 5.1. SELDI-TOF-MS of **11a** is shown below:

**
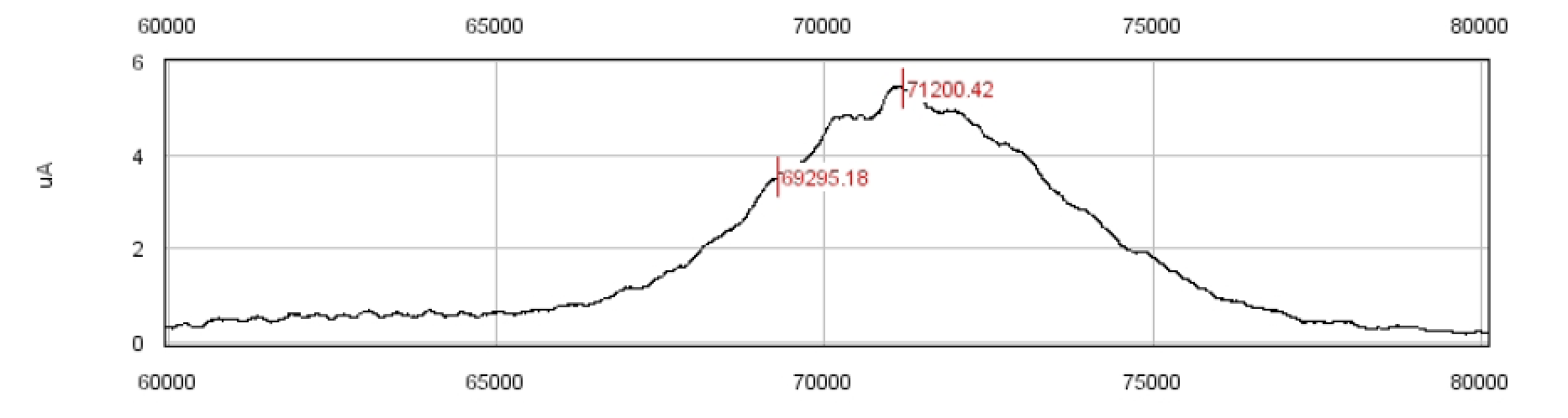
**

**12a:**

Antigen **12**^S6^ (5.0 mg, 6.6 μmol) was treated with 3,4-Dimethoxy-3-cyclobutene-1,2-dione (5.3 mg, 37.3 μmol) in pH 7 buffer for 16h to give squarate derivative (4.55 mg, 80%) as a white solid. HRMS-ESI: m/z [M+H]^+^ calcd for C_31_H_48_N_2_O_24_P: 863.2335; found: 863.2328. Methyl squarate derivative of **12** (0.47 mg, 0.545 μmol) was conjugated to BSA (4.75 mg, 0.0715 μmol) in pH 9 buffer to give 2.96 mg of white fluffy solid conjugate **12a** (59%). Estimated antigen to protein ratio: 4.9. SELDI-TOF-MS of **12a** is shown below (the signal at 66,282.32 Da is a signal of BSA added as internal standard into the SELDI sample):

**
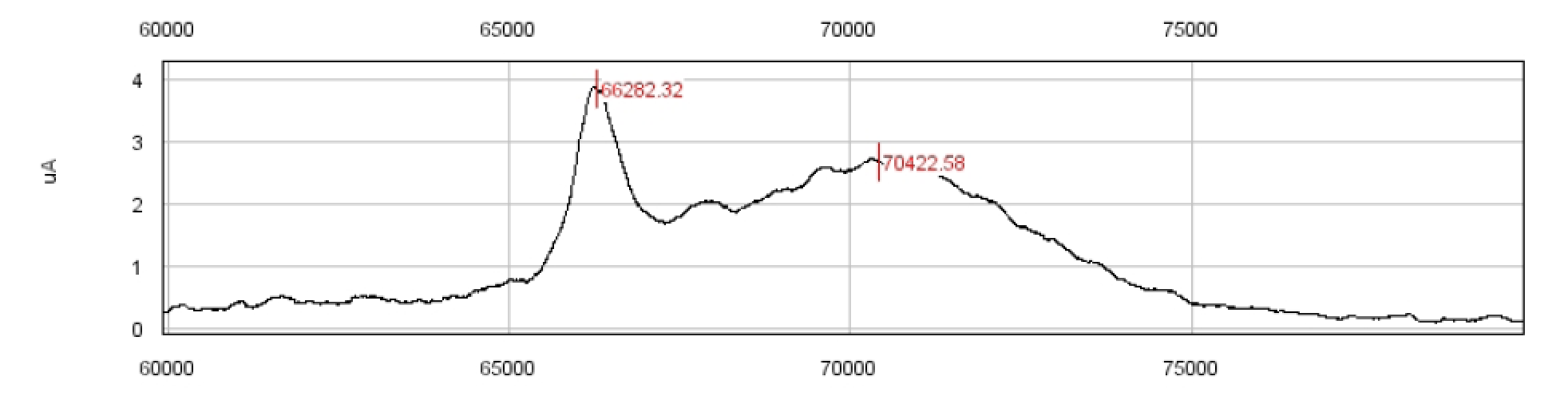
**

**13a:**

Antigen **13**^S7^ (6.05 mg, 7.23 μmol) was treated with 3,4-Dimethoxy-3-cyclobutene-1,2-dione (10.3 mg, 72.3 μmol) in pH 7 buffer for 15h to give squarate derivative (5.17 mg, 76%) as a white solid. HRMS-ESI: m/z [M+H]^+^ calcd for C_37_H_60_N_2_O_24_P: 947.3274; found: 947.3275. Methyl squarate derivative of **13** (0.37 mg, 0.391 μmol) was conjugated to BSA (3.76 mg, 0.0566 μmol) in pH 9 buffer to give 3.82 mg of white fluffy solid conjugate **13a** (96%). Estimated antigen to protein ratio: 4.4. SELDI-TOF-MS of **13a** is shown below:

**
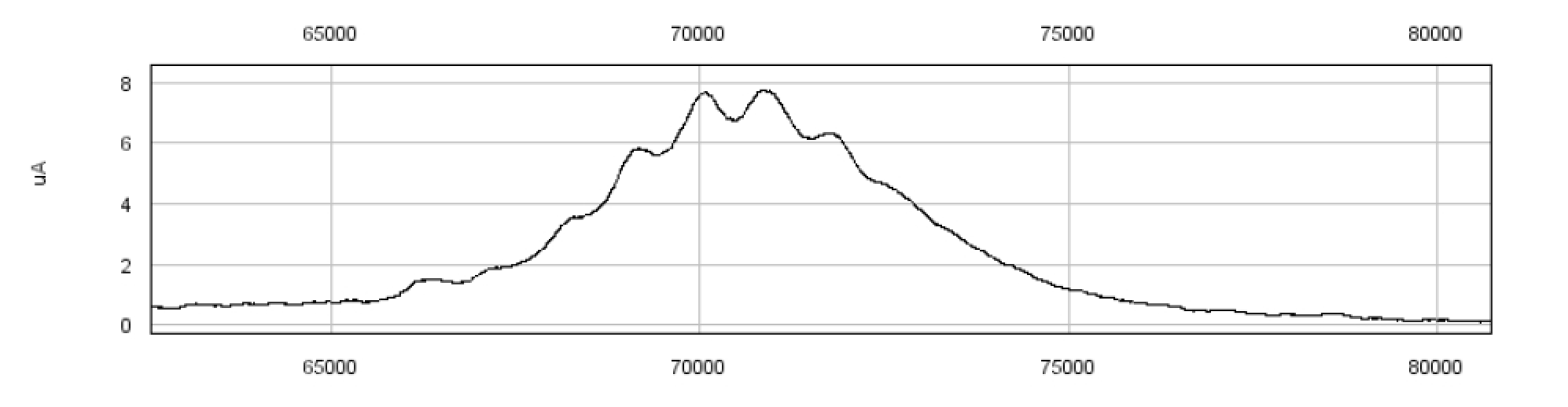
**

**
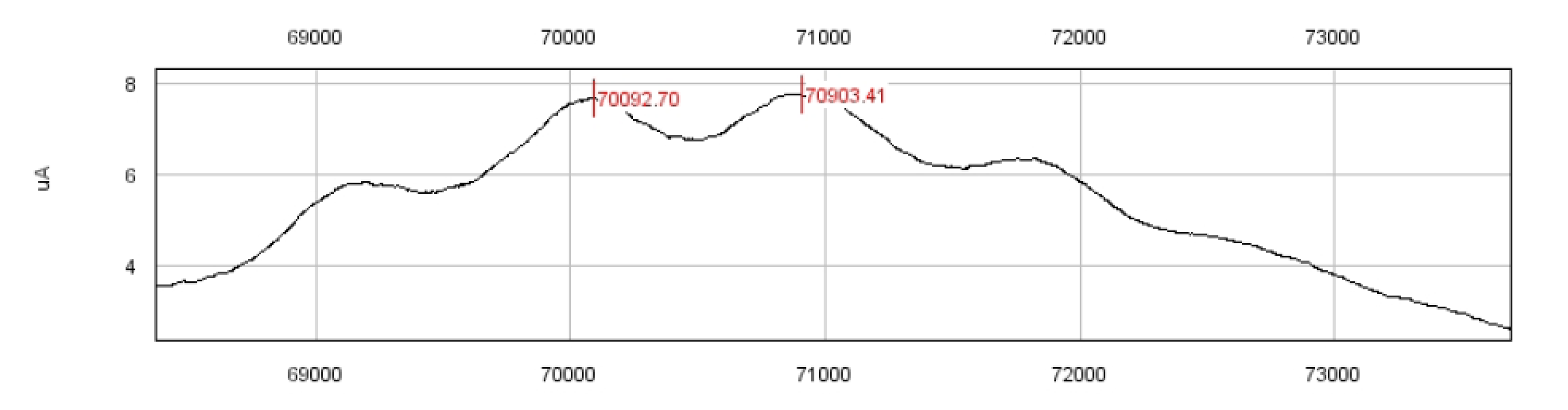
**

**14a:**

Antigen **14**^S8^ (6.0 mg, 8.49 μmol) was treated with 3,4-Dimethoxy-3-cyclobutene-1,2-dione (6.0 mg, 42.2 μmol) in pH 7 buffer for 14h to give squarate derivative (4.45 mg, 64%) as a white solid. Methyl squarate derivative of **14** (0.95 mg, 1.16 μmol) was conjugated to BSA (6.0 mg, 0. 0903 μmol) in pH 9 buffer to give 5.5 mg of white fluffy solid conjugate **14a** (85%). Estimated antigen to protein ratio: 5.1. SELDI-TOF-MS of **14a** is shown below:

**
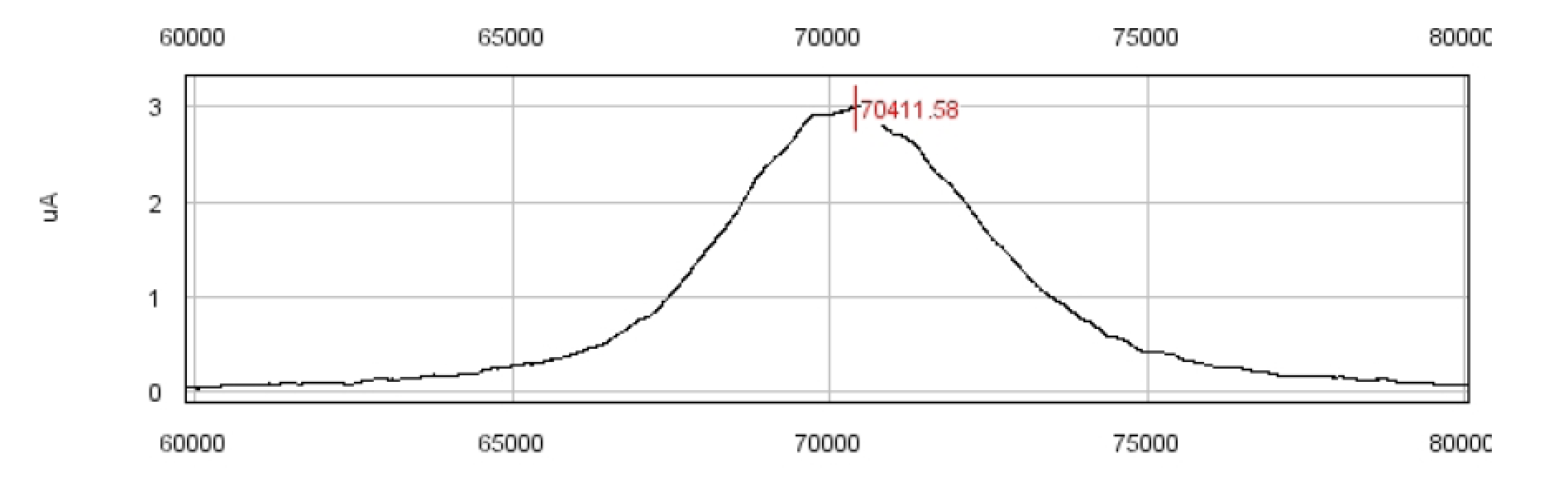
**

**15a:**

Antigen **15**^S7^ (5.7 mg, 8.1 μmol) was treated with 3,4-Dimethoxy-3-cyclobutene-1,2-dione (11.5 mg, 81.0 μmol) in pH 7 buffer for 6h to give squarate derivative (5.86 mg, 89%) as a white solid. HRMS-ESI: m/z [M+H]^+^ calcd for C_31_H_50_N_2_O_21_P: 817.2644; found: 817.2640. Methyl squarate derivative of **15** (0.7 mg, 0.857 μmol) was conjugated to BSA (9.69 mg, 0.146 μmol) in pH 9 buffer to give 9.75 mg of white fluffy solid conjugate **15a** (97%). Estimated antigen to protein ratio: 3.5. SELDI-TOF-MS of **15a** is shown below:

**
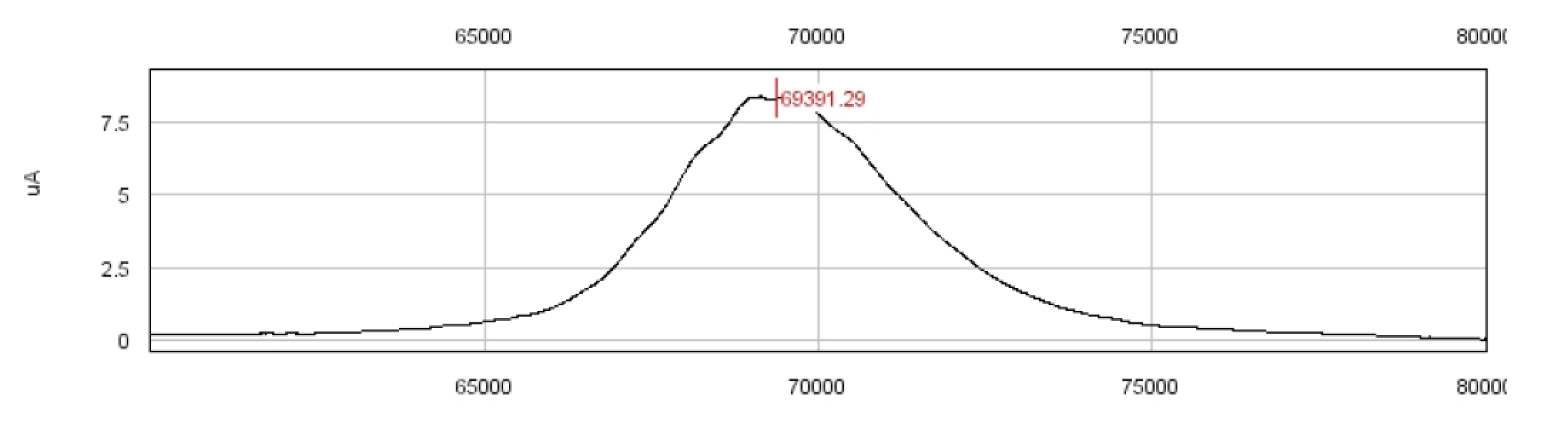
**

**16a:**

Antigen **16**^S7^ (7.6 mg, 13.2 μmol) was treated with 3,4-Dimethoxy-3-cyclobutene-1,2-dione (10.6 mg, 74.6 μmol) in pH 7 buffer for 16h to give squarate derivative (5.9 mg, 65%, purified by HPLC eluted with MeCN:MeOH:H_2_O/64:18:18) as a white solid. HRMS-ESI: m/z [M+Na]^+^ calcd for C_25_H_39_N_2_O_18_NaP: 709.1833; found: 709.1822. Methyl squarate derivative of **16** (0.3 mg, 0.437 μmol) was conjugated to BSA (4.85 mg, 0.073 μmol) in pH 9 buffer to give 4.0 mg of white fluffy solid conjugate **16a** (79%). Estimated antigen to protein ratio: 5.0. SELDI-TOF-MS of **16a** is shown below (the signal at 66,375.50 Da is a signal of BSA added as internal standard into the SELDI sample):

**
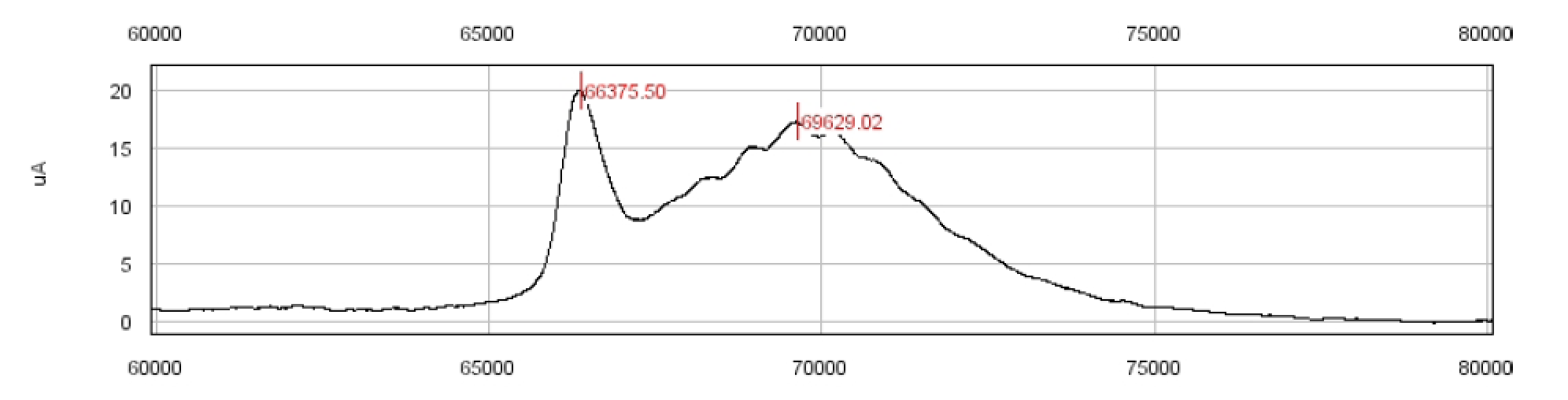
**

**17a:**

Drierite (266 mg) was added into a solution of antigen 1**7**^S9^ (266 mg, 0.53 mmol) in anhydrous EtOH (5.3 mL). After stirring for 60 min, 3,4-diethoxy-3-cyclobutene-1,2-dione (98 μL, 0.663 μmol) and KHCO_3_ (265 mg, 2.65 mmol) were added and the reaction mixture was stirred at room temperature for 6.5h (with occasionally sonication to increase solubility of KHCO_3_). After TLC confirmed that the reaction was finished, the reaction mixture was filtered, concentrated and purified by flash chromatography to give squarate derivative (305 mg, 92%) as a white solid. ESI-MS: m/z [M-H]^-^ 626. Ethyl squarate derivative of **17** (0.52 mg, 0.83 μmol) was conjugated to BSA (9.1 mg, 0.137 μmol) in pH 9 buffer to give 8.95 mg of white fluffy solid conjugate **17a** (94%). Estimated antigen to protein ratio: 5.0. SELDI-TOF-MS of **17a** is shown below:

**
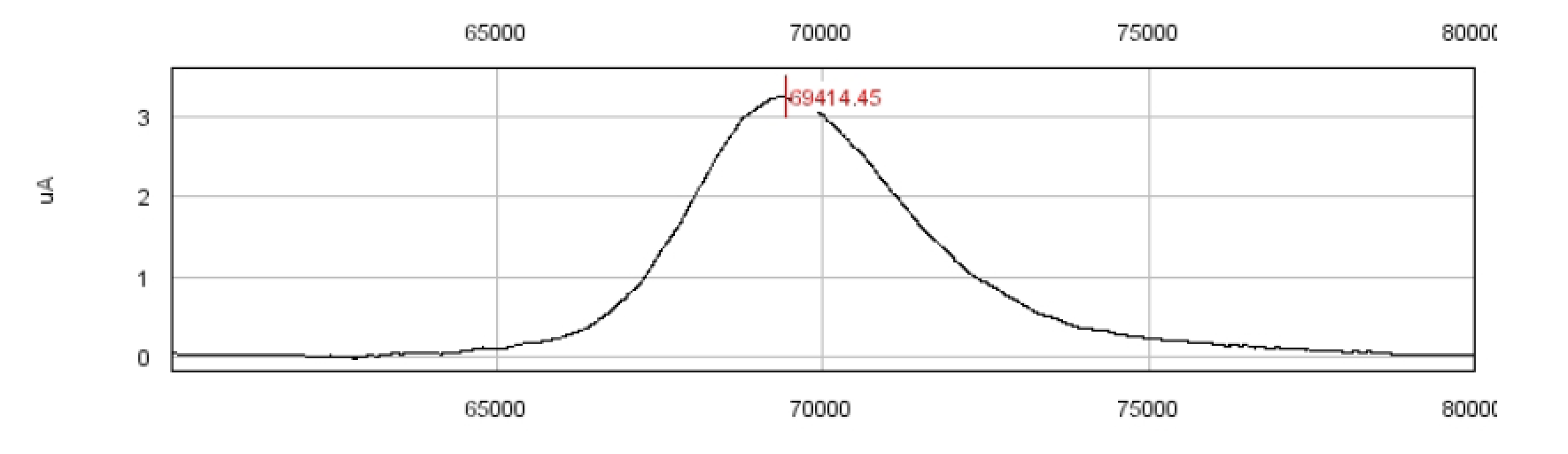
**

**18a:**

Antigen **18**^S8^ (10.0 mg, 19.5 μmol) was treated with 3,4-Dimethoxy-3-cyclobutene-1,2-dione (7.2 mg, 50.7 μmol) in pH 7 buffer for 14h to give squarate derivative (3.1 mg, 26%, low yield due to purification by preparative TLC plate) as a white solid. Methyl squarate derivative of **18** (0.6 mg, 0.964 μmol) was conjugated to BSA (8.0 mg, 0.12 μmol) in pH 9 buffer to give 8.0 mg of white fluffy solid conjugate **18a** (95%). Estimated antigen to protein ratio: 6.0. SELDI-TOF-MS of **15a** is shown below:

**
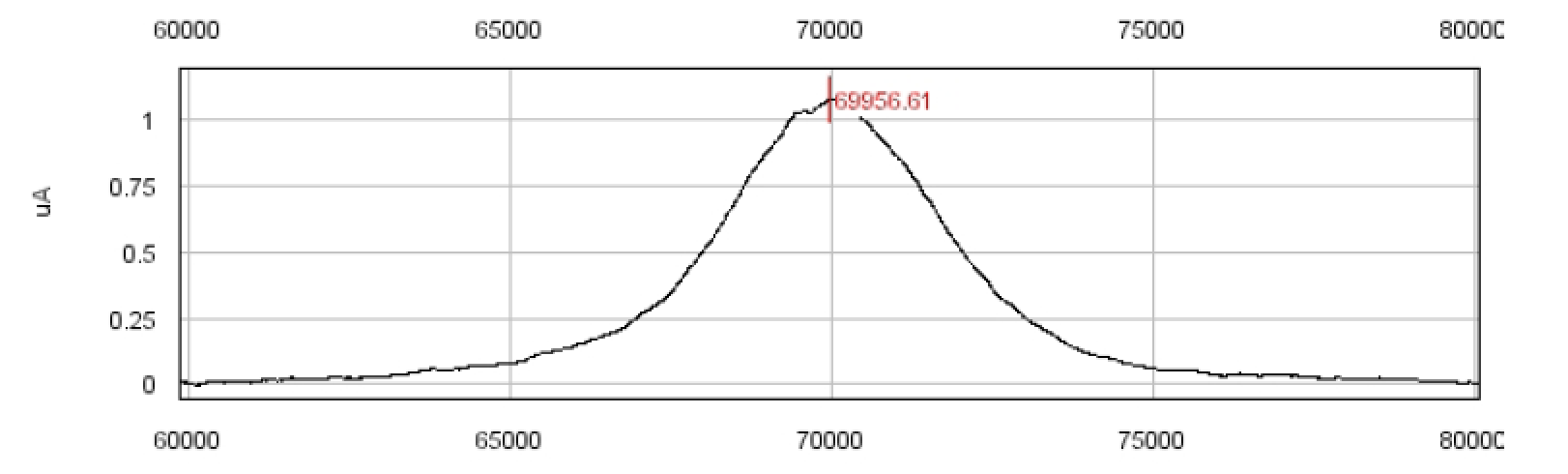
**

**1b:**

Methyl squarate derivative of **1**^S4^ (1.28 mg, 0.481 μmol) was conjugated to rTT-Hc (2.5 mg, 0.0481 μmol) in pH 9 buffer to give 3.1 mg of white fluffy solid conjugate **1b** (96%). Estimated antigen to protein ratio: 5.7. SELDI-TOF-MS of **1b** is shown below:

**
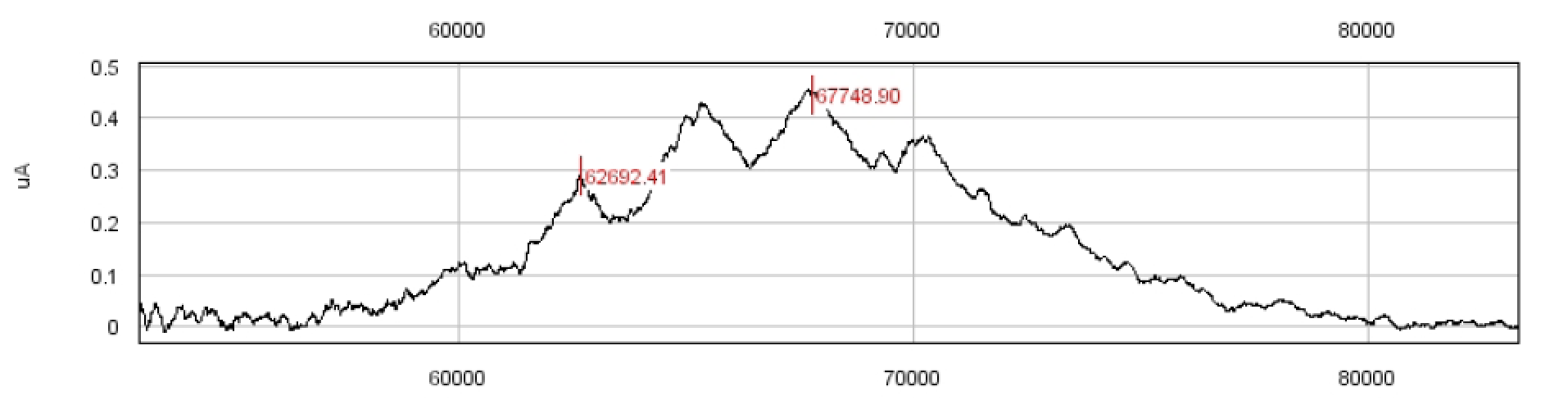
**

**3b:**

Methyl squarate derivative of **3**^S4^ (0.95 mg, 0.355 μmol) was conjugated to rTT-Hc (1.85 mg, 0.0355 μmol) in pH 9 buffer to give 2.3 mg of white fluffy solid conjugate **3b** (97%). Estimated antigen to protein ratio: 5.7. SELDI-TOF-MS of **3b** is shown below:
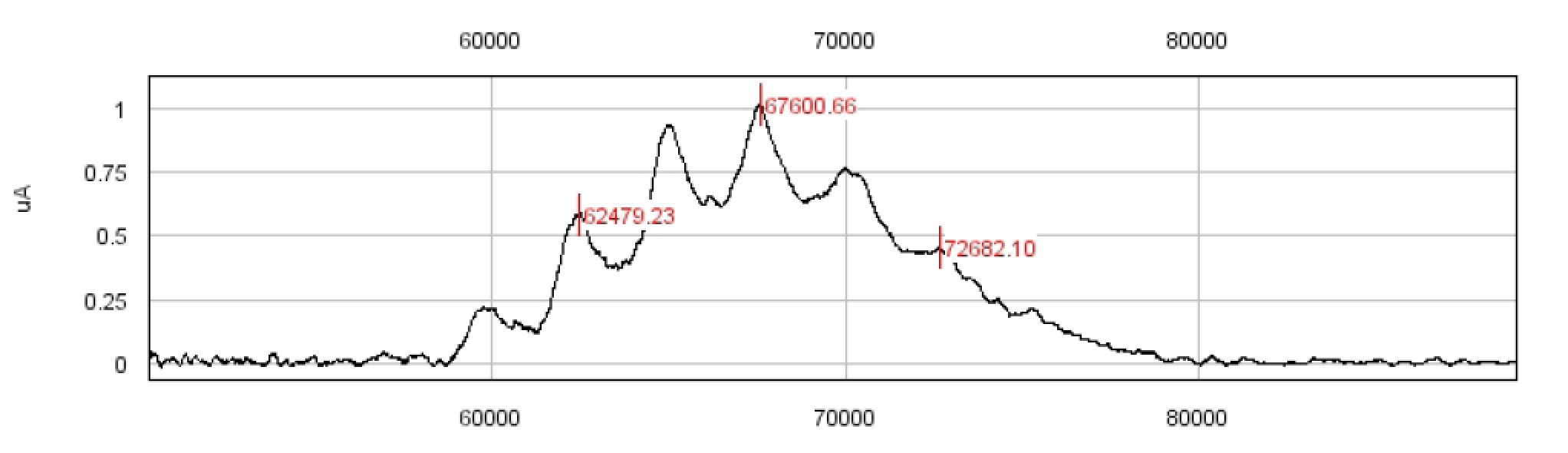


**4b:**

Methyl squarate derivative of **4**^S5^ (1.19 mg, 0.91 μmol) was conjugated to rTT-Hc (7.85 mg, 0.151 μmol) in pH 9 buffer to give 8.1 mg of white fluffy solid conjugate **4b** (94%). Estimated antigen to protein ratio: 4.1. SELDI-TOF-MS of **4b** is shown below:


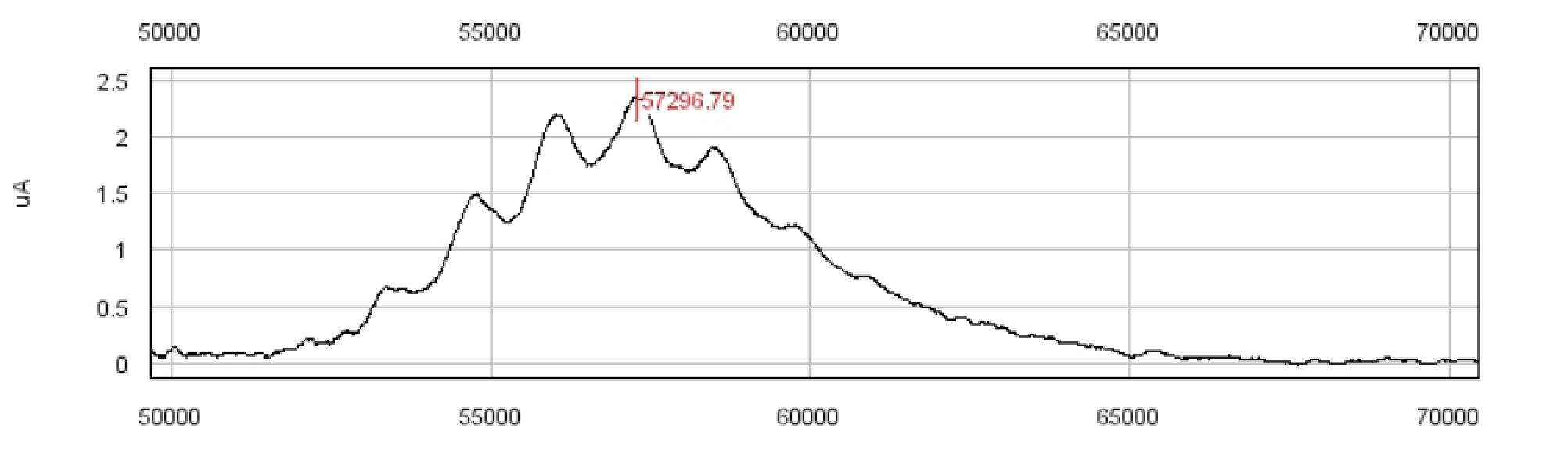


**5b:**

Methyl squarate derivative of **5**^S5^ (1.21 mg, 0.91 μmol) was conjugated to rTT-Hc (7.91 mg, 0.152 μmol) in pH 9 buffer to give 8.39 mg of white fluffy solid conjugate **5b** (97%). Estimated antigen to protein ratio: 4.0. SELDI-TOF-MS of **5b** is shown below:


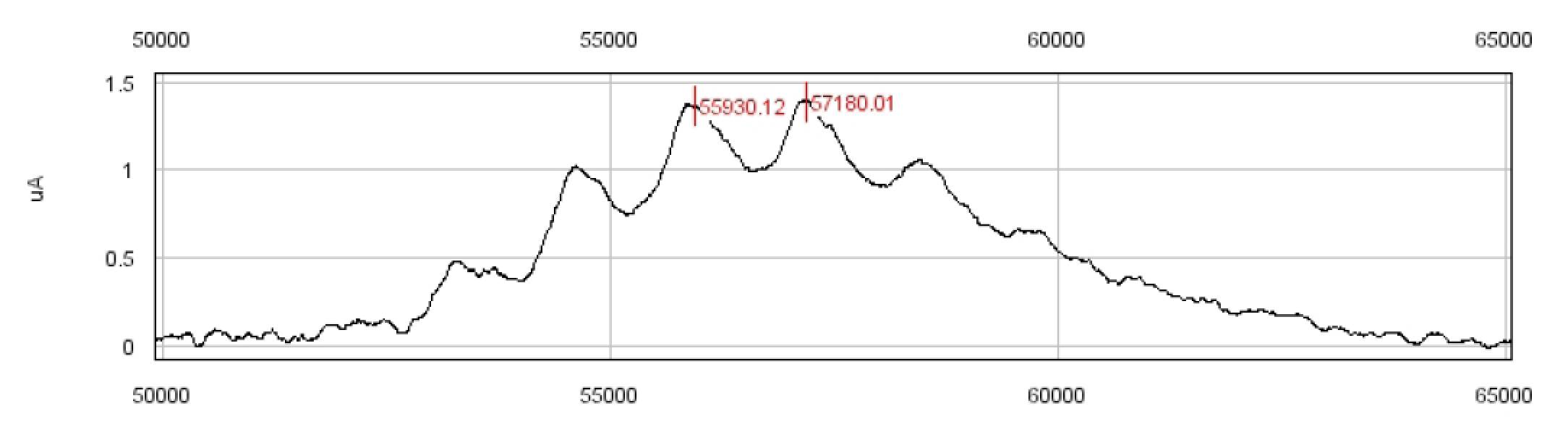


**7b:**

Methyl squarate derivative of **7** (1.2 mg, 1.07 μmol) was conjugated to rTT-Hc (9.23 mg, 0.178 μmol) in pH 9 buffer to give 9.72 mg of white fluffy solid conjugate **7b** (95%). Estimated antigen to protein ratio: 5.1. SELDI-TOF-MS of **7b** is shown below:


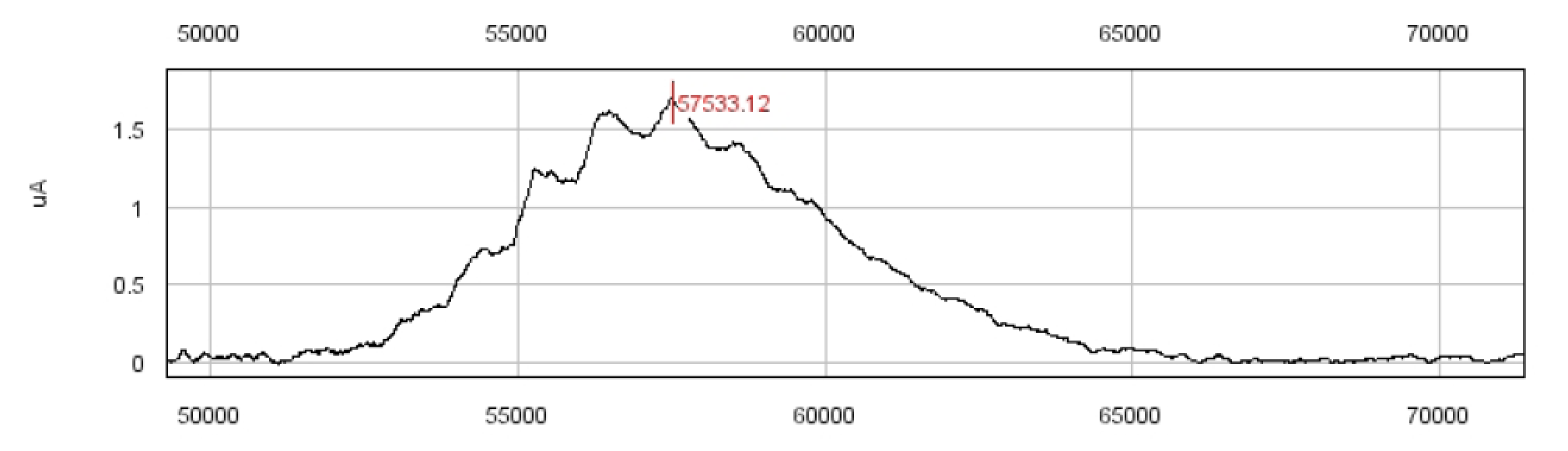


**8b:**

Methyl squarate derivative of **8** (1.21 mg, 1.07 μmol) was conjugated to rTT-Hc (9.23 mg, 0.178 μmol) in pH 9 buffer to give 10.4 mg of white fluffy solid conjugate **8b** (100%). Estimated antigen to protein ratio: 5.4. SELDI-TOF-MS of **8b** is shown below:


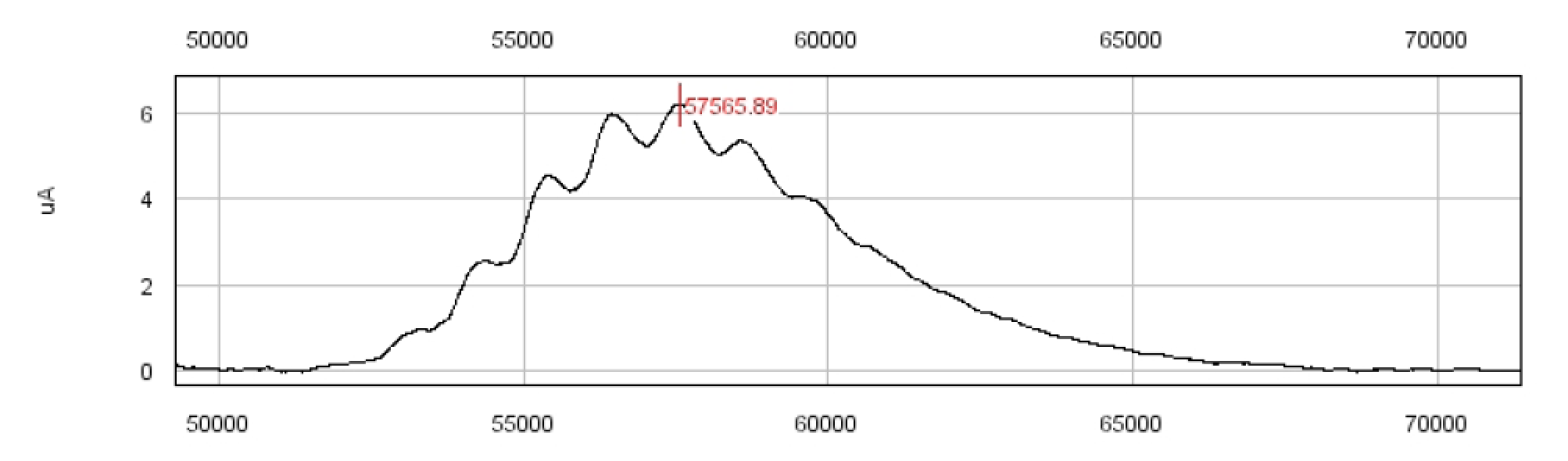


**13b:**

Methyl squarate derivative of **13** (1.0 mg, 1.09 μmol) was conjugated to rTT-Hc (9.15 mg, 0.177 μmol) in pH 9 buffer to give 9.32 mg of white fluffy solid conjugate **13b** (92%). Estimated antigen to protein ratio: 5.8. SELDI-TOF-MS of **13b** is shown below:


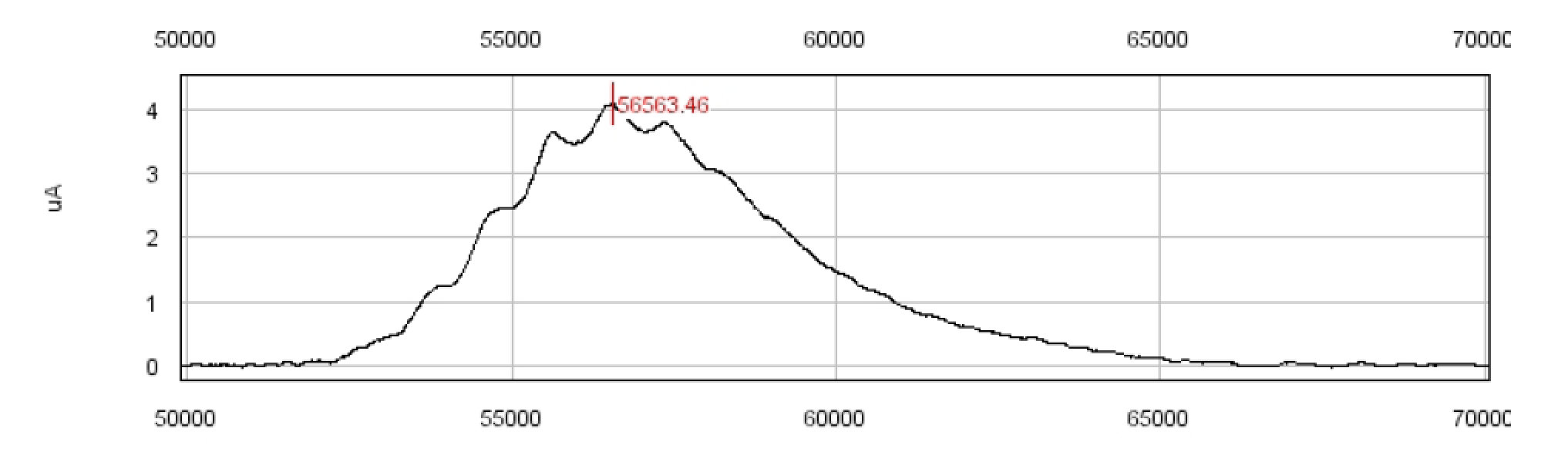


**Supplemental Method References:**

S1. Kováč P, Xu P. 2017. Controlled and highly efficient preparation of carbohydrate-based vaccines: squaric acid chemistry is the way to go. Carbohydr Chem. 42:83–115.

S2. Pfister HB, Lu X, Soliman SE, Kováč P. 2019. Conjugation of synthetic oligosaccharides to proteins by squaric acid chemistry. p. 77–88. In: Methods in Molecular Biology. Vol. 1954. Humana Press Inc.

S3. Xu P, Kováč P. 2019. Direct conjugation of bacterial polysaccharides to proteins by squaric acid chemistry. p. 89–98. In: Methods in Molecular Biology. Vol. 1954. Humana Press Inc.

S4. Xu P, Korcová J, Baráth P, Čížová A, Valáriková J, Qadri F, Kelly M, O’Connor RD, Ryan ET, Bystrický S, Kováč P. 2019. Isolation, purification, characterization and direct conjugation of the lipid A-free lipopolysaccharide of *Vibrio cholerae* O139. Chem - A Eur J. 25(56):12946–56.

S5. Lu X, Pfister HB, Soliman SE, Kováč P. 2018. O-Specific Polysaccharide of *Vibrio cholerae* O139: Improved synthesis and conjugation to BSA by squaric acid chemistry. European J Org Chem. 2018(23):2944–57.

S6. Lu X, Kováč P. 2016. Chemical synthesis of the galacturonic acid containing pentasaccharide antigen of the O-specific polysaccharide of *Vibrio cholerae* O139 and its five fragments. J Org Chem. 81(15):6374–94.

S7. Ruttens B, Saksena R, Kováč P. 2007. Synthesis of phosphorylated, conjugation-ready di-, tri- and tetrasaccharide fragments of the O-specific polysaccharide of *V. cholerae* O139. European J Org Chem. 2007(26):4366–75.

S8. Soliman SE, Kováč P. 2015. Stereoselective syntheses of the conjugation-ready, downstream disaccharide and phosphorylated upstream, branched trisaccharide fragments of the O-PS of *Vibrio cholerae* O139. J Org Chem. 80(10):4851–60.

S9. Ruttens B, Kováč P. 2006. Synthesis of a phosphorylated disaccharide fragment of the O-specific polysaccharide of *Vibrio cholerae* O139, functionalized for conjugation. Helv Chim Acta. 89(2):320–32.
